# Detection and characterization of protein methylation in bacteriophages and their host, *Cellulophaga baltica*, during infection

**DOI:** 10.1128/msystems.00012-26

**Published:** 2026-05-18

**Authors:** Andrew J. Stai, Cristina Howard-Varona, Marion Urvoy, Marissa R. Gittrich, Matthew B. Sullivan, Robert L. Hettich

**Affiliations:** 1 Biosciences Division, Oak Ridge National Laboratory 6146, Oak Ridge, Tennessee, USA; 2 The Bredesen Center for Interdisciplinary Research and Graduate Education, The University of Tennessee 4292, Knoxville, Tennessee, USA; 3 Department of Microbiology, The Ohio State University 2647, Columbus, Ohio, USA; 4 Center of Microbiome Science, The Ohio State University 2647, Columbus, Ohio, USA; 5 Department of Civil, Environmental and Geodetic Engineering, The Ohio State University 2647, Columbus, Ohio, USA; Yale University, New Haven, Connecticut, USA; Shenzhen University, Shenzhen, China; Huazhong University of Science and Technology, Wuhan, China; University of Bologna, Bologna, Italy

**Keywords:** bacteriophages, proteomics, protein methylation, phage infection, protein post-translational modification

## Abstract

Bacteria and their viruses (bacteriophages or phages) are in a dynamic “arms race” that balances predation and resistance, using various strategies to achieve dominance. To better understand these strategies, we infected the marine model bacterium *Cellulophaga baltica* with three previously characterized phages (phi18:1, phi18:4, and phi38:1), each with diverse genome types and infection efficiencies. Mass spectrometry-based proteomics measurements of the molecular signatures of the phage infection processes revealed that the percentage of peptides identified as methylated was approximately doubled in phage-infected cells (virocells) compared to uninfected cells, with a significantly increased frequency of host protein methylation observed in the early stage of the infections. Additionally, phage proteins displayed methylation in both virocells and free virions, neither of which has been previously reported. This suggests that protein post-translational methylation may be an important regulator in the phage-bacterial infection process. To explore this further, we assessed methylation patterns in virocells versus uninfected cells at a global level, as well as for a selected set of host proteins with known importance to phage infection, including elongation factor thermo unstable (EF-Tu), chaperone DnaK, and gliding motility proteins. Collectively, our results expand the molecular details of the phage-host arms race by highlighting dynamic protein methylation signatures in phage infection.

**IMPORTANCE:**

The battle for survival between bacteria and their associated phages has broad ecological implications due to the evolutionary pressure each exerts on the other, as well as the unique phenotypes presented by phage-infected bacteria. The various mechanisms through which bacteria and their phages wage this battle is an active area of exploration. Here, we used the marine model bacterium *Cellulophaga baltica* and three of its phages to identify and examine a previously unreported method of regulation in phage-infected bacteria: protein methylation. We report the novel finding of methylation of phage proteins and demonstrate this methylation is not just restricted to viral proteins within the cell but is also found in free virions outside the cell. Furthermore, our study opens up future, protein-specific mechanistic investigation by highlighting virocell-unique methylation patterns on several host proteins with known importance to phage infection.

## INTRODUCTION

Bacteria and the viruses that infect them (phages) are the two most abundant biological entities on Earth and are in a constant battle of survival and predation. This has significant consequences for microbial diversity and evolution (1) and potentially vast impacts on ecosystem outputs and functioning (2, 3). Through viral lysis, horizontal gene transfer, and metabolic reprogramming, viruses can both shunt (remineralize) (4) and/or shuttle (sink or remove) organic matter (5), influencing the flow of nutrients in diverse ecosystems. For example, in the oceans, global ocean carbon flux from surface to deep waters is best predicted by viral abundances, even over prokaryotic or eukaryotic abundances (2), demonstrating the great impact of viral activities. Additionally, at least one in five cells in the oceans (6) are virus-infected (termed “virocells”), and the transcript, protein, and metabolite reprogramming undergone during infection results in a unique metabolic footprint on the environment for virocells compared to their uninfected counterparts (7). Thus, virocells warrant consideration in population, community, ecosystem, and Earth system models, with recent interest focused on elucidating mechanisms of virus-host interaction biology. Such studies are critical to better understand the arms race, whereby each virus and host evolve new strategies to gain advantages over the other (8) that likely underpins the functioning of most ecosystems.

Despite being very ancient (9), this arms race is constantly evolving, with foundational discoveries flooding recent literature. Bacteria have numerous opportunities to counter phage infection, as they can (i) evolve spontaneous mutations to modify cell membrane receptors and prevent phage adsorption (10); (ii) target inserted viral genomes for degradation (via restriction-modification systems that modify and thereby protect host nucleic acids against restriction proteins, or CRISPR-Cas systems that “record” past infection nucleic acids for future CRISPR system targeting) (11); or (iii) employ abortive infection systems (self-induced cell death or dormancy) (11). Because of their biotechnological and ecological importance, there has been an explosion of research into phage defense systems that has revealed dozens of new systems by which bacteria defend against phages, including through chemical defenses, co-opting intracellular communication, as well as many unknown mechanisms (12, 13).

Protein modifications are also being increasingly appreciated for their role in viral defense systems. In eukaryotes, host cells defend against viral infection by tagging viral proteins with post-translational modifications (PTMs) to target them for degradation (14, 15) or disrupt their enzymatic activity (14). Furthermore, host cells can also modify their own proteins to assist in antiviral immune responses (14, 15). Given the arms race dynamics, however, it is not surprising that eukaryotic viruses have evolved counter-defenses that leverage PTMs for their own advantage, with PTMs promoting critical points of the viral life cycle, such as its attachment, genome replication, and release (16, 17).

Similarly to eukaryotes and their viruses, PTMs are also actively used in the arms race between bacteria and their phages. Bacteria utilize PTMs in defending themselves against phages, such as by modifying their own proteins to induce cell death (18) or prevent phage adsorption (19). On the other hand, phages can target or use PTMs on host proteins to take over the cell, such as by interfering with PTM-mediated induced cell death (18) or by preventing host-induced disruption of translation (20). In addition to PTMs on host proteins being observed during phage infection, PTMs on phage proteins have also been seen. These include post-translational cleavage of T4 capsid proteins (21), ubiquitin-like protein conjugation to interfere with virion assembly of tailed coliphages (22), and acetylation and phosphorylation of diverse marine phage proteins making up the virion, including major capsid proteins (MCPs) (23).

Despite the known importance of PTMs in phage infection, there are still many PTMs whose roles have not been reported in this context, one of which is protein methylation. Protein methylation is most often reported on the side-chain nitrogens of lysines and arginines (24) and increases its residue’s hydrophobicity, potentially altering the structure of the protein and thereby its function. Additionally, protein methylation can serve as part of a motif recognized by protein “methyl readers” that preferentially bind to methylated forms of proteins, influencing the protein interaction network of the methylated proteins. Protein methylation plays important roles in various common bacterial cellular processes, such as motility (25), adhesion (26), and stress response (27). In addition to this, protein methylation has been seen as active and important in eukaryotic viral infections (16, 28–30). However, protein methylation has not been reported in phage infection, and its prevalence and role in prokaryotic virocells remain unknown.

To elucidate the range and dynamics of protein methylation in bacteria-phage interactions throughout and across phage infections, we utilized high-resolution mass spectrometry to characterize the proteome of a common marine bacterium, *Cellulophaga baltica* strain #18 (Cba18), over the early, middle, and late stages of three different previously characterized phage infections (31, 32). The infecting phages had different genomic properties and infection characteristics on Cba18, specifically phi18:1 (dsDNA, efficient infection), phi18:4 (ssDNA, efficient infection), and phi38:1 (dsDNA, inefficient infection) (32–34). Proteomics analysis revealed increased levels of protein methylation during the infections, including shortly after phage adsorption. In a more detailed examination, we found novel evidence of methylation occurring on phage proteins and even being maintained in the free virion. Differences among virocells and uninfected cells for overall host protein methylation and protein-specific methylation were also identified, including proteins with previously known relationships to phage infection, such as the GTPase elongation factor thermo unstable (EF-Tu) and chaperone DnaK.

## RESULTS AND DISCUSSION

### Protein methylation is readily detected and elevated in virocells relative to uninfected cells

**Fig 1:**
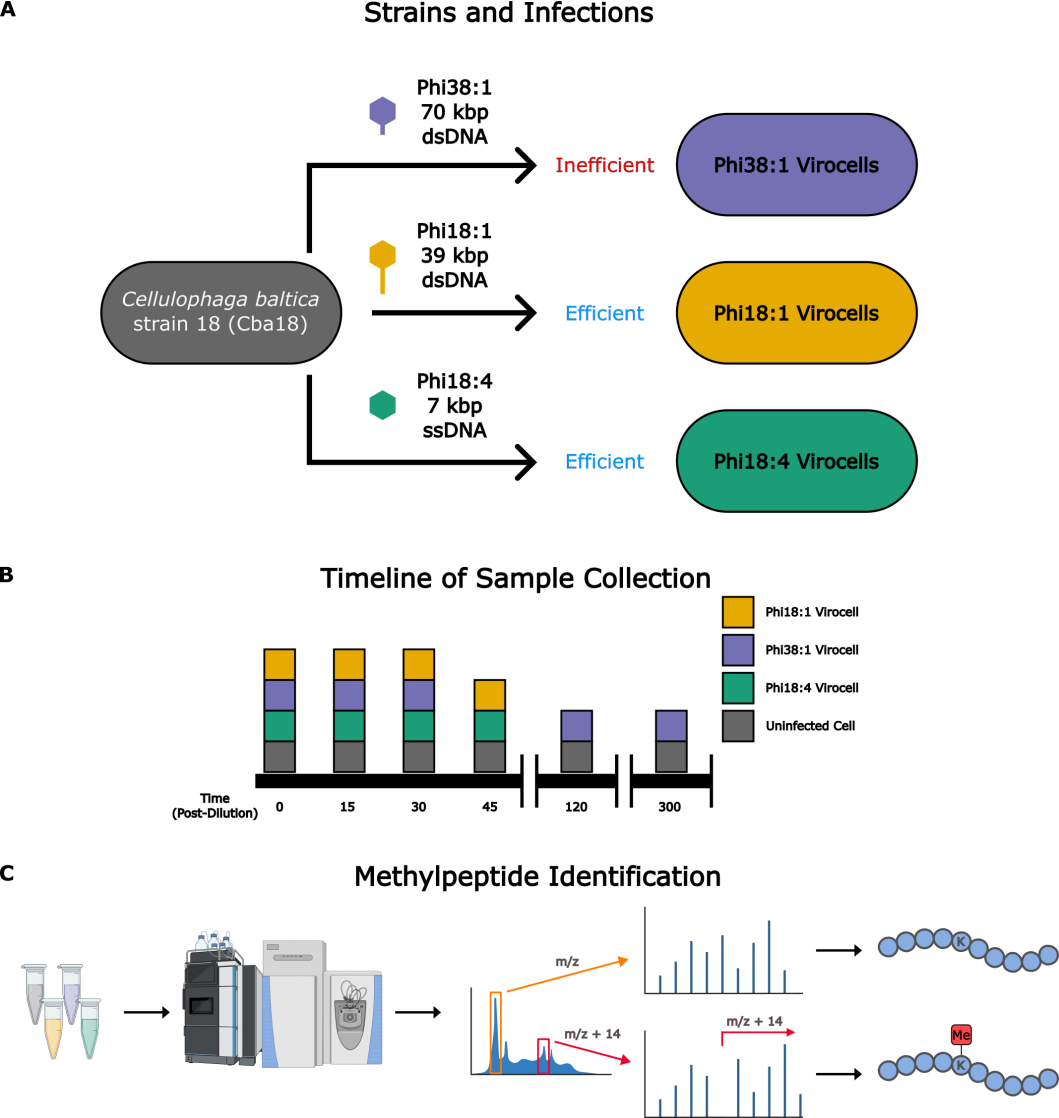
Overview of the experimental design with uninfected cells in gray, phi38:1 virocells in purple, phi18:1 virocells in yellow, and phi18:4 virocells in green. (**A**) Depiction of bacterial and viral strains and their resulting infections. (**B**) Timeline of sample collection used for virocell and uninfected cell proteomics data. (**C**) Workflow of methylpeptide identification from collected samples run through an LC-MS setup and analyzed for mass shifts in both precursor and fragmentation scans to identify methylpeptides, with the monomethyl group denoted by “Me” in red on a lysine residue within a peptide.

An overview of the experimental design is given in Fig. 1. Briefly, Cba18 cells were inoculated with one of the three phages with different genome properties and infection efficiencies (Fig. 1A). Samples of the infected cells were then collected at various time points after dilution, along with samples of an uninfected cell culture (Fig. 1B). All sampling time points fell within or near the estimated latent period of the respective bacterial infections (phi18:1, 65 minutes [32]; phi18:4, 45 minutes [32]; and phi38:1, >240 minutes [31]), ensuring that protein or peptide abundance changes were not a result of an increase in phage particles. Proteins were isolated from the samples and digested into peptides, followed by untargeted proteomic analysis by mass spectrometry, which identified methylated peptides (methylpeptides) by a mass shift corresponding to the number of methyl groups attached to the peptide (Fig. 1C).

Untargeted proteome measurements of Cba18 across uninfected and phage-infected virocells detected a large fraction (3,334/3,971) of the bacterium’s predicted proteins. Similarly, the majority of the phages’ predicted proteins were detected in their respective virocell samples (phi18:1, 57/65; phi38:1, 76/101; phi18:4, 12/13). The total number of proteins identified in each individual sample showed similar levels, ranging from 2,000 to 2,600 (Table S1). In the process of examining the resulting proteome data sets, we noticed a significantly elevated amount of protein methylation in the virocell samples compared to uninfected cell samples (*n =* 52 virocell samples and 24 uninfected cell samples; two-sided Mann-Whitney *U*; *P =* 2.89e-8), with the percentage of peptides detected as methylated in virocells being on average double or more than that of uninfected cells (Table 1; Table S2; uninfected cells, 0.6%; phi18:1 virocells, 1.9%; phi38:1 virocells, 2%; phi18:4 virocells, 1.4%). For a high-level inspection of virocell protein methylation, we quantified the number and proportion of methylpeptides identified across the uninfected host cells and the three virocells; the results are summarized in Table 1. This suggested methylation as a possible mechanism of control in the virocell systems. Additionally, as support for this protein post-translational modification, we detected three annotated protein methyltransferases (Ga0325142_11476, Ga0325142_114013, and Ga0325142_114045) in the measured proteome of the host bacteria, verifying the protein methylation capacity in this system, though the real number of protein methyltransferases encoded by Cba18 is likely higher, given that nearly one-quarter of the detected proteome had no functional annotation, and several other methyltransferases had ambiguous annotations as to their target molecules (DNA, RNA, protein, etc.). The significantly elevated detection of methylated peptides prompted a deeper look into protein methylation in these virocells.

**TABLE 1:** Number and portion of identified methylated peptides across time and conditions^*a*^

Sample	No. of methylpeptides	No. of peptides	Peptides methylated (%)
Uninfected cells T0	202 ± 20	18,921 ± 483	1.1 ± 0.1
Uninfected cells T15	35 ± 5	15,575 ± 950	0.2 ± 0
Uninfected cells T30	34 ± 1	15,664 ± 442	0.2 ± 0
Uninfected cells T45	275 ± 100	18,172 ± 564	1.5 ± 0.5
Uninfected cells T120	103 ± 59	17,670 ± 519	0.6 ± 0.3
Uninfected cells T300	42 ± 1	17,160 ± 130	0.2 ± 0
**Uninfected cells (avg)**	**115 ± 104**	**17,193 ± 1,358**	**0.6 ± 0.5**
Phi18:1 virocells T0	516 ± 17	19,244 ± 153	2.7 ± 0.1
Phi18:1 virocells T15	246 ± 91	17,634 ± 292	1.4 ± 0.5
Phi18:1 virocells T30	68 ± 25	14,651 ± 1,362	0.5 ± 0.1
Phi18:1 virocells T45	527 ± 9	16,544 ± 1,248	3.2 ± 0.3
**Phi18:1 virocells (avg)**	**339 ± 199**	**17,018 ± 1,916**	**1.9 ± 1.1**
Phi38:1 virocells T0	315 ± 5	16,580 ± 469	1.9 ± 0.1
Phi38:1 virocells T15	215 ± 23	16,238 ± 141	1.3 ± 0.1
Phi38:1 virocells T30	400 ± 14	15,123 ± 257	2.6 ± 0.1
Phi38:1 virocells T120	304 ± 15	14,345 ± 544	2.1 ± 0.2
Phi38:1 virocells T300	333 ± 15	14,862 ± 133	2.2 ± 0.1
**Phi38:1 virocells (avg)**	**313 ± 61**	**15,429 ± 915**	**2 ± 0.5**
Phi18:4 virocells T0	209 ± 47	13,204 ± 2,010	1.6 ± 0.1
Phi18:4 virocells T15	156 ± 41	13,107 ± 1,324	1.2 ± 0.2
Phi18:4 virocells T30	188 ± 13	13,490 ± 216	1.4 ± 0.1
Phi18:4 virocells T45	162 ± 19	11,369 ± 1,986	1.4 ± 0.2
**Phi18:4 virocells (avg.)**	**179 ± 39**	**12,792 ± 1,773**	**1.4 ± 0.2**

^*a*^ Specific time point values were generated by averaging across all biological replicates (*n* = 4) for each sample, with standard deviations of the replicates given. Averages of all time points for a given condition (*n* = 24, *n* = 16, *n* = 20, *n* = 16 for uninfected cells, phi18:1 virocells, phi38:1 virocells, and phi18:4 virocells, respectively) are in bold, with standard deviations of the samples given.

**Fig 2:**
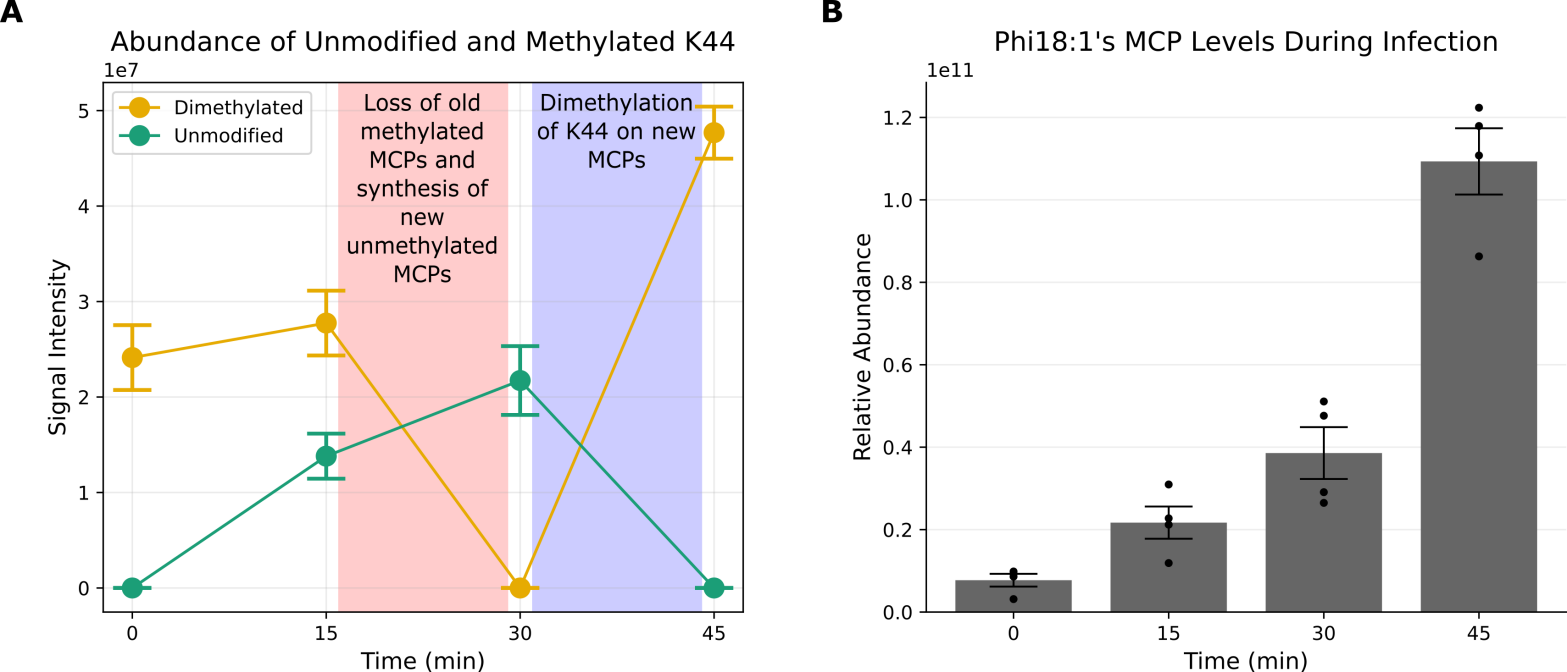
Phi18:1’s major capsid protein (MCP) methylation and relative abundance over time. (**A**) Comparison of unmodified (green) and dimethylated (yellow) peptides that encompass K44 of phi18:1’s gp49 MCP. Red highlighted region covers time in which predicted MCPs from previous infection are being degraded or falling off the host, and new MCPs are being synthesized. Blue highlighted region covers time in which newly synthesized MCPs are predicted to be dimethylated at K44 for protein localization or virion formation. Error bars representing the SEM of biological replicates (=4) are shown with vertical brackets. (**B**) Bar chart showing relative abundance of phi18:1’s MCP gp49 during infection. Relative protein abundance was estimated by summing peptide intensities for each time point. Gray bar heights represent averages of biological replicates (=4), and replicates are plotted in black. SEM error bars are shown with vertical brackets.

Methylpeptides were in general of lower abundance than unmodified peptides, with the mean signal intensity being about fourfold lower for methylpeptides compared to unmodified peptides (Fig. S1A). Additionally, there was an observed correlation of the ability to detect methylation of a given protein with that protein’s abundance. While there were certainly abundant proteins with no detected methylation, proteins with higher abundance were more likely to have detected methylation than proteins with lower abundance (Fig. S1B). One important note, however, is that changes in methylpeptide abundance often did not correlate with changes in the corresponding protein’s abundance (see Fig. 2 as an example). This highlights the role of methylation as a dynamic modification that provides protein regulation beyond their simple abundance changes.

### Phage proteins are methylated in virocells, and some maintain methylation between infections in free virions

After cataloging the number of methylpeptides identified from both host and infecting phage proteins in each virocell, we sought to examine methylation of phage proteins specifically. Methylation of viral proteins has been previously reported for eukaryotic viruses (16, 30) but never for phage proteins. In all three studied virocells here, methylation of both lysine and arginine residues was identified on phage proteins, with 84 manually validated methylation events (see Supplemental text for definition) on 33 phage proteins (phi18:1, 15; phi38:1, 14; and phi18:4, 4) (Table 2). Around one-third of phage proteins with detected methylation had no functional annotations, which is not unexpected, since 70% of all known phage proteins have no functional annotations (35).

**TABLE 2:** Methylation events on phage proteins in virocells^*a*^

Phage	Gene accession	Annotation	Location and number of methyl groups
Phi18:1	gp01	DNA methylase	**K122(D)**, **K237(D)**, **R250(D)**, **R267(D)**
Phi18:1	gp09	Hypothetical protein	K21(D), **K69(**M**/D)**, R103(D), **K119(**M**/D)**
Phi18:1	gp10	Hypothetical protein	**R75(D)**
Phi18:1	gp16	Hypothetical protein	R63(D)
Phi18:1	gp33	Phage terminase small subunit	K150(D)
Phi18:1	gp35	Phage portal protein	K256(D)
Phi18:1	gp38	Hypothetical protein	K91(D)
Phi18:1	gp40	DNA methylase	K54(D), K61(D)
Phi18:1	gp42	Hypothetical protein	K51(D)
Phi18:1	gp46	Hypothetical protein	K27(D), R38(D)
Phi18:1	gp49	Phage major capsid protein	K15(D), K34(D), **K44(D)**, K78(D), K107(D), R152(D), K169(D), K185(M/D), K207(D), K314(M/D), R360(D), K384(M/D), R390(D)
Phi18:1	gp52	Putative phage tail fiber	K586(D)
Phi18:1	gp54	Hypothetical protein	K125(D)
Phi18:1	gp55	Phage tail protein	K59(D), R107(D)
Phi18:1	gp61	Structural protein	K251(M)
Phi38:1	gp003	Hypothetical protein	K106(D)
Phi38:1	gp009	Putative mitogen-activated protein kinase	R32(M)
Phi38:1	gp011	Hypothetical protein	**K19(D)**, **R29(D)**, **K45(D)**, **K68(D)**
Phi38:1	gp021	Peptidase	K244(D)
Phi38:1	gp022	Putative ABC transporter	**R287(D)**
Phi38:1	gp028	Putative 4-phytase	**R31(D)**, **K39(D)**
Phi38:1	gp035	Putative oxidoreductase	**K16(D)**, K30(D), **R78(D)**, **K105(D)**
Phi38:1	gp039	Putative 26S proteasome regulatory subunit	**K125(D)**, K171(M)
Phi38:1	gp045	Putative 5′-nucleotidase deoxy(pyrimidine) cytosolic type C protein	**K68(D)**
Phi38:1	gp047	DNA polymerase	K598(T)
Phi38:1	gp054	Chaperonin GroEL	K121(D), K133(D), **K241(D)**, K321(D), **K361(D)**
Phi38:1	gp057	Hypothetical protein	K89(M)
Phi38:1	gp067	Structural protein, putative major capsid protein	K359(M)
Phi38:1	gp081	RyR domain containing protein	K78(M)
Phi18:4	gp04	Phage major capsid protein	**K9(**M**/D)**, R27(D), **K39(D)**, K46(D)
Phi18:4	gp05	Structural protein	R22(M/D), K41(D), K66(D)
Phi18:4	gp06	Structural protein	K54(D), K69(D), K86(D), R111(D)
Phi18:4	gp09	Structural protein	K57(D), K92(D), K100(D), K107(D)

^*a*^ Identified methylation state for a lysine (K) or arginine (R) residue is denoted by “(M)” for monomethylation, “(D)” for dimethylation, or “(T)” for trimethylation. Multiple identified methylation states for a residue are denoted by “/.” Residue and methylation states in bold denote the methylation event was detected at T0. All methylation events were automatically identified by Proteome Discoverer and manually reevaluated by inspection of MS2 spectra.

Methylation of all three phages’ major capsid proteins (MCPs) was observed. Capsid proteins are known to exhibit a variety of post-translational modifications in eukaryotic viral infections (28, 36–38), including methylation (28, 29, 36). Eukaryotic viral capsid methylation has been observed to affect virion assembly (28) as well as capsid protein localization within the cell (29). In most cases for our study, capsid methylation seemed to be an infrequent event, with the signal intensity (see Table S3 for definition) of many MCP methylpeptides being one-tenth or less than that of their unmethylated counterparts. However, there were a few methylation events that were more prevalent and time dependent. On phi18:1’s MCP (gene gp49), all identified peptides covering lysine (K) 44 were dimethylated at that residue at T0 and T45, while at T15 the portion of those peptides with K44 methylation decreased to ~67%, and K44 methylpeptides were not detected at all at T30 (Fig. 2A). One possible explanation of this pattern is that the MCP is dimethylated at K44 in preparation for or to assist with virion assembly and maintains this dimethylation in the free virion to the next host infection. The MCP is then degraded and/or falls off the host cell shortly after infection, is created anew with no methylation for the next round of viral replication, and is finally methylated again in preparation for virion assembly (Fig. 2A). This would explain why all phi18:1 MCPs had K44 dimethylation at T0 and T45 but had decreased and no portion dimethylated at T15 and T30, respectively, as the phage had begun creating new, unmethylated capsid proteins by T15, reflected in the increased measured protein levels (Fig. 2B). The prevalence of the K44 dimethylation event suggests a meaningful role this event plays in the functioning of the protein, where dimethylation may be required for proper assembly of the virion complex, similar to how methylation of capsid proteins for certain eukaryotic viruses assists in their virion formation (28, 29). As phi18:1 is not predicted to contain any methyltransferases in its genome, this suggests an additional reliance of phi18:1 on the host’s methyltransferases to methylate its capsid protein, demonstrating another unique way in which phages make use of their hosts’ cellular machinery for their own benefit.

As mentioned above, a portion of phage proteins (12/33) had methylation observed at T0 (see footnote in Table 2) in each phage infection, which corresponds to when the phages have recently adsorbed to the cell. None of these phages are known to encode methyltransferases, indicating that these proteins are likely either (i) being methylated by host protein methyltransferases very early on in the infection or (ii) have maintained methylation during the free virion stage—as seen previously with phosphorylation and acetylation (23)—that occurred in their previous host. There was evidence for both hypotheses, as some methylation events were only found early in infection and were not detected in the late stages of infection (indicative of the former), while other methylation events were found at the beginning of infection, disappeared, and then later were detected prior to phage lysis of the cell (indicative of the latter). The cause of this methylation disappearance is unknown but may be the result of a variety of factors, such as protein turnover or the activity of an unannotated protein demethylase encoded by Cba18. Regarding the possibility of the proteins being demethylated, none of the current gene annotations indicate the presence of a protein demethylase in the Cba18 genome. However, a large portion of the host proteins detected in any sample (653/3,334) had no annotation, leaving this as a possibility. In support of possible demethylase activity, gene Ga0325142_111104 encoded by Cba18 and detected in our proteomics data shares a 77% amino acid identity with a predicted lysine demethylase from another bacterium (UniProt ID A0ABM9NWM9 [39], *Tenacibaculum platacis*) in the same family as Cba18, Flavobacteriaceae. This gene is annotated in the Cba18 genome as producing a cupin-like protein. The cupin superfamily is known to contain a family of histone demethylases (40), lending further support to this protein’s identity as a demethylase, though whether it is responsible for any of these disappearing methylation events remains to be examined.

To test whether some of these methylation events on phage proteins are being maintained in the free virion stage, MS proteomic analysis was conducted on samples with free phage virions released after Cba18 lysis (see Supplemental text). Nine, two, and two phage proteins were detected as methylated for phi18:1, phi38:1, and phi18:4 virions, respectively (Table 3). Nearly all methylated proteins detected were structural proteins, including phi18:1’s MCP gp49. This is consistent with the observation of structural proteins often being the most abundant proteins in a phage particle, frequently having many copies making up the capsid (41, 42), giving the highest likelihood of detecting methylation, if present in those proteins (Fig. S1B). Ten of the total 28 methylation events detected in the phage-free virions were also seen in virocells (see footnote in Table 3). There were two methylation events (dimethylation of K69 on gp9 of phi18:1 and dimethylation of K39 on gp4 of phi18:4) that were found at the beginning and end of infection, as well as in the free virions of the phages (Table S4), indicating that some of the methylation observed at T0 in the virocells may have occurred in the previous infection and was maintained through the free virion stage of the phage. While the function of phi18:1’s gp9 is unknown, gp4 of phi18:4 is the phage’s MCP. Given that phi18:4’s MCP was methylated at K39 at the beginning and end of infection as well as in the free virion—all time points when phi18:4’s MCP would be forming or composing the capsid—one possible function of this methylation event is that it may assist in forming the viral capsid complex, similar to the proposed function of phi18:1’s MCP K44 methylation discussed above. However, in the free virion, the K39 methylpeptide’s signal intensity was a small fraction (<1%) of the unmodified peptide’s signal intensity, indicating any effect provided by the methylation is likely not necessary for the functioning of the protein.

**TABLE 3:** Methylation events on phage proteins in free virions^*a*^

Phage	Gene accession	Annotation	Location and number of methyl groups
Phi18:1	gp08	Putative AAA family ATPase	K310(D)
Phi18:1	gp09	Hypothetical protein	**K69(M/D)**, K144(M/D)
Phi18:1	gp16	Hypothetical protein	**R63(D)**
Phi18:1	gp35	Phage portal protein	K244(D), **K256(D)**, K293(D), R342(D)
Phi18:1	gp36	Phage minor capsid protein	K104(D)
Phi18:1	gp49	Phage major capsid protein	**K185(M/D)**, **K384(M/D)**
Phi18:1	gp50	Head-tail connector protein	K168(D)
Phi18:1	gp52	Putative phage tail fiber	R23(D), K108(D), R580(D)
Phi18:1	gp59	Structural protein	K684(D)
Phi38:1	gp054	Chaperonin GroEL	K27(D)
Phi38:1	gp067	Putative major capsid protein	**K359**(**M**/D), K385(M)
Phi18:4	gp04	Major capsid protein	**K39(D)**, K186(M/D)
Phi18:4	gp05	Structural protein	K92(D)

^*a*^ Identified methylation state for a lysine (K) or arginine (R) residue is denoted by “(M)” or “(D)” for monomethylation and dimethylation, respectively. Multiple identified methylation states for a residue are denoted by “/.” Residue and methylation states in bold denote the methylation event was also detected in the respective virocells. All methylation events were automatically identified by Proteome Discoverer and manually reevaluated by inspection of MS2 spectra.

Altogether, these results demonstrate that phage proteins, especially structural proteins, are targeted for methylation and can maintain their methylation between hosts in the free virion stage.

### Phage infection has significant effects on host Cba18’s protein methylation levels

**Fig 3:**
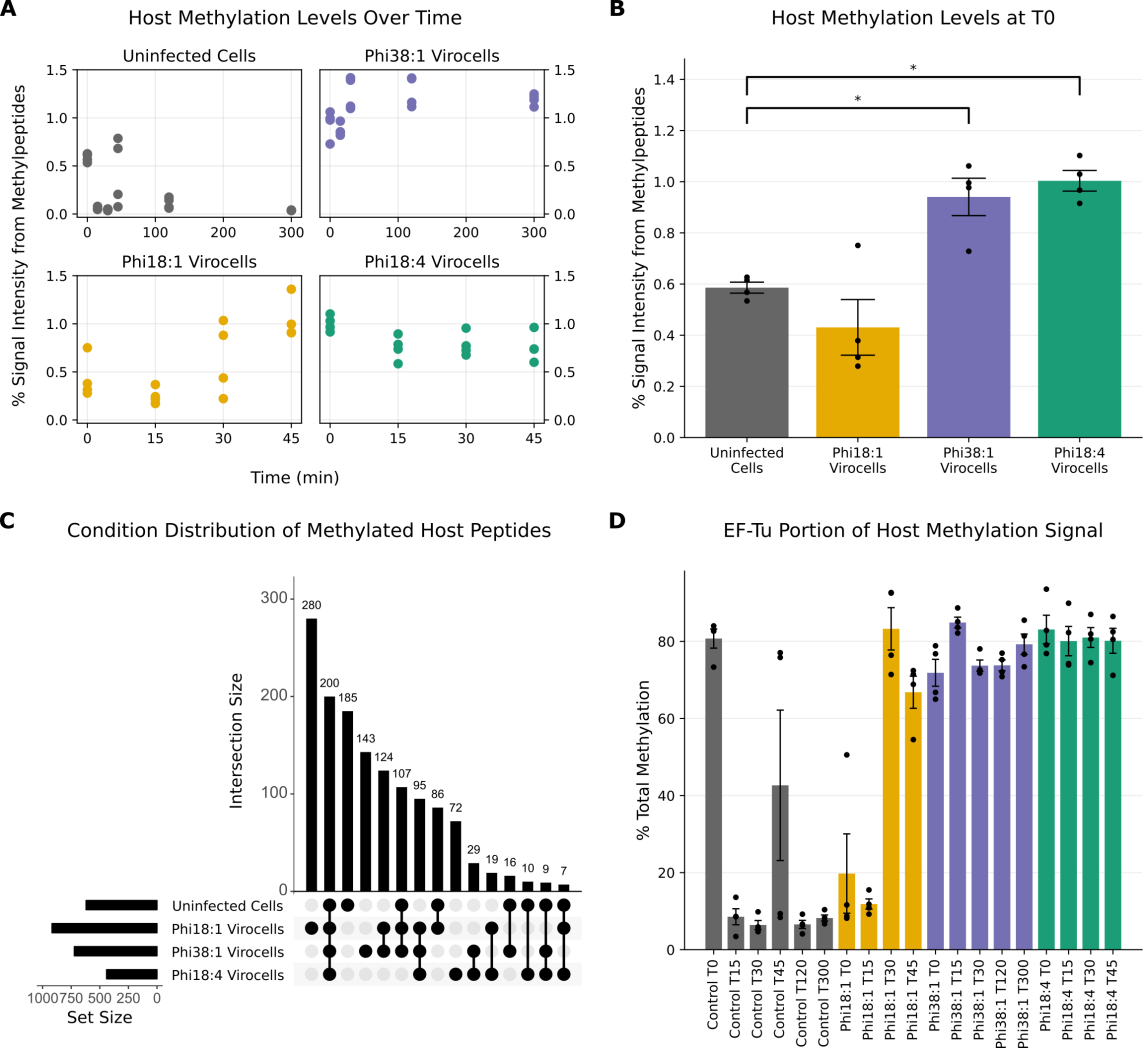
Intensity and distribution of methylation on host Cba18 proteins. (**A**) Scatterplots showing the percentage of the total measured signal intensity coming from host methylpeptides at each time point in each condition, with four biological replicates per time point. The percentage of signal intensity was calculated by summing signal intensity from host methylated peptides and dividing that by the sum of signal intensity from all host peptides. (**B**) Bar chart showing the percentage of total measured signal intensity coming from host methylpeptides (calculated same as in panel A) at T0. Bar heights represent averages of biological replicates (*n* = 4), with replicates plotted in black. Horizontal brackets above bars indicate significance (two-sided Mann-Whitney *U* test), with “*” for adjusted *P* values = 0.043. SEM error bars are shown with vertical brackets. (**C**) UpSet plot showing distribution of detected methylated host peptides among conditions and intersections of conditions, where connected black circles signify the above bar number of methylated peptides were found in and only in those specific conditions. The set size for each condition gives the total number of host methylpeptides identified in that condition across all time points. (**D**) Bar chart showing the percentage of host’s total methylation signal from EF-Tu methylpeptides. Percentage of total methylation was calculated by summing signal intensities of EF-Tu methylpeptides and dividing by the summed signal intensity of all host methylpeptides. Bar heights represent averages of biological replicates (*n* = 4), with replicates plotted in black. SEM error bars are shown with vertical brackets. Sample names beginning with “control” denote uninfected cells, and sample names beginning with “phi” denote virocells of respective phages.

After characterizing phage protein methylation, we examined the impact of infection on the host’s overall protein methylation levels. We sought to characterize the effect the different phage infections have on the total abundance of host protein methylation within the cell by calculating the percentage of total measured host peptide signal intensity coming from Cba18 methylpeptides for every condition and time point (Fig. 3A). Significantly elevated percentages of methylation compared to uninfected cells were observed for both phi38:1 and phi18:4 virocells (*n* = 4, two-sided Mann-Whitney *U*, *P* = 0.043) in the early stage of infection at T0 (Fig. 3B). Given that T0 is when most phages have just adsorbed to the cell, this indicates that the host may be detecting the presence of phage, either in the extracellular environment or early in the phage’s infection, and increasing protein methylation as part of a stress response. This is supported by Cba18 having previously been seen to quickly upregulate its stress response genes in response to phi38:1 on a similar time scale (34). While phi38:1 and phi18:4 virocells had significantly elevated levels of methylation early in infection compared to uninfected cells, phi18:1 virocell methylpeptide levels were more similar to uninfected cells at T0, with on average a non-significant decrease compared to uninfected cells (*n* = 4, two-sided Mann-Whitney *U*, *P* = 0.343) (Fig. 3B). This was unexpected, as phi18:1 virocells at T0 had the second most identified methylpeptides of any time point across all samples (Table 1), with many of these being unique (Fig. 3C), indicating that many of these unique methylation events occurring in phi18:1 virocells are in low abundance. Much of these differences in abundance of methylation signal can be explained by a single protein: the GTPase EF-Tu. In most samples, methylation of EF-Tu makes up the majority of the measured methylation signal (Fig. 3D). However, for phi18:1 virocells in early infection, EF-Tu’s portion of methylation is decreased near 10-fold compared to uninfected cells. Therefore, it seems that phi18:1 virocells may have a larger number of unique (Fig. 3C) but less abundant (Fig. 3B) methylation events to regulate the cell compared to phi38:1 and phi18:4 virocells, which display more abundant, shared methylation events such as with methylation of EF-Tu. In summary, these results indicate that the host proteome is differentially methylated in response to different phage infections, with phi18:1 virocells displaying more unique methylation events but phi38:1 and phi18:4 virocells having more abundant protein methylation on the whole, mostly arising from extensive methylation of EF-Tu.

### Prevalent and variable methylation of elongation factor-Tu protein may influence phage ability to replicate

After examining global methylation patterns of the host, we next directed our efforts toward analyzing methylation events on specific host proteins of interest. First, we decided to examine proteins with prevalent methylation. Proteins involved in protein metabolism (ribosomal proteins, elongation factor proteins, etc.) and energy (ATP synthases, dehydrogenases, etc.) made up the largest portion of detected methylation events across all samples (Fig. S2A). Specifically, ribosomal proteins and EF-Tu were frequent targets of methylation, comprising nearly a quarter (23%) of all detected methylation events on host proteins (Fig. S2B). This was expected because (i) both are known to be in high abundance due to their important role in protein synthesis, making methylation detection easier (Fig. S1B), and (ii) both are known to be targeted for methylation in bacteria (43–47).

While several ribosomal proteins in bacteria have been seen to be fairly constitutively methylated (44, 45), EF-Tu’s methylation is more context specific. Currently, only two sites on EF-Tu are reported to be methylated in bacteria: K5 in *Pseudomonas aeruginosa* that is trimethylated to aid in bacterial cell attachment to the host cells (43) and K56 in *Escherichia coli* that has been seen to be variably mono- and dimethylated in different bacterial growth phases (46) as well as in response to stress and nutrient deprivation (47). The effect of K56 methylation is not entirely known, but it has been observed to result in attenuated GTPase activity hypothesized to be caused by the methylation interfering with conformation effects induced on EF-Tu by tRNA binding (46). This attenuated GTPase activity is then predicted to result in a slower and more accurate translation process (46). In addition to these observations in bacteria, methylation of eEF1A, the eukaryotic homolog of EF-Tu, is important for formation of a viral replication complex for tombusvirus (48). Furthermore, EF-Tu is already known to be an important player in some phage infections, where it serves in phage replication as a subunit of the Qβ replicase (49), and is also used by infected bacteria as a degradation target to inhibit protein translation and induce cell death (50, 51). Because of such evidence, we suspected that EF-Tu’s methylation may be a source of functional control utilized by the host or the infecting phage to gain an advantage in the battle of infection. Given all this and its large contribution to the methylation signal (Fig. 3D), we decided to investigate EF-Tu’s methylation in greater detail.

**Fig 4:**
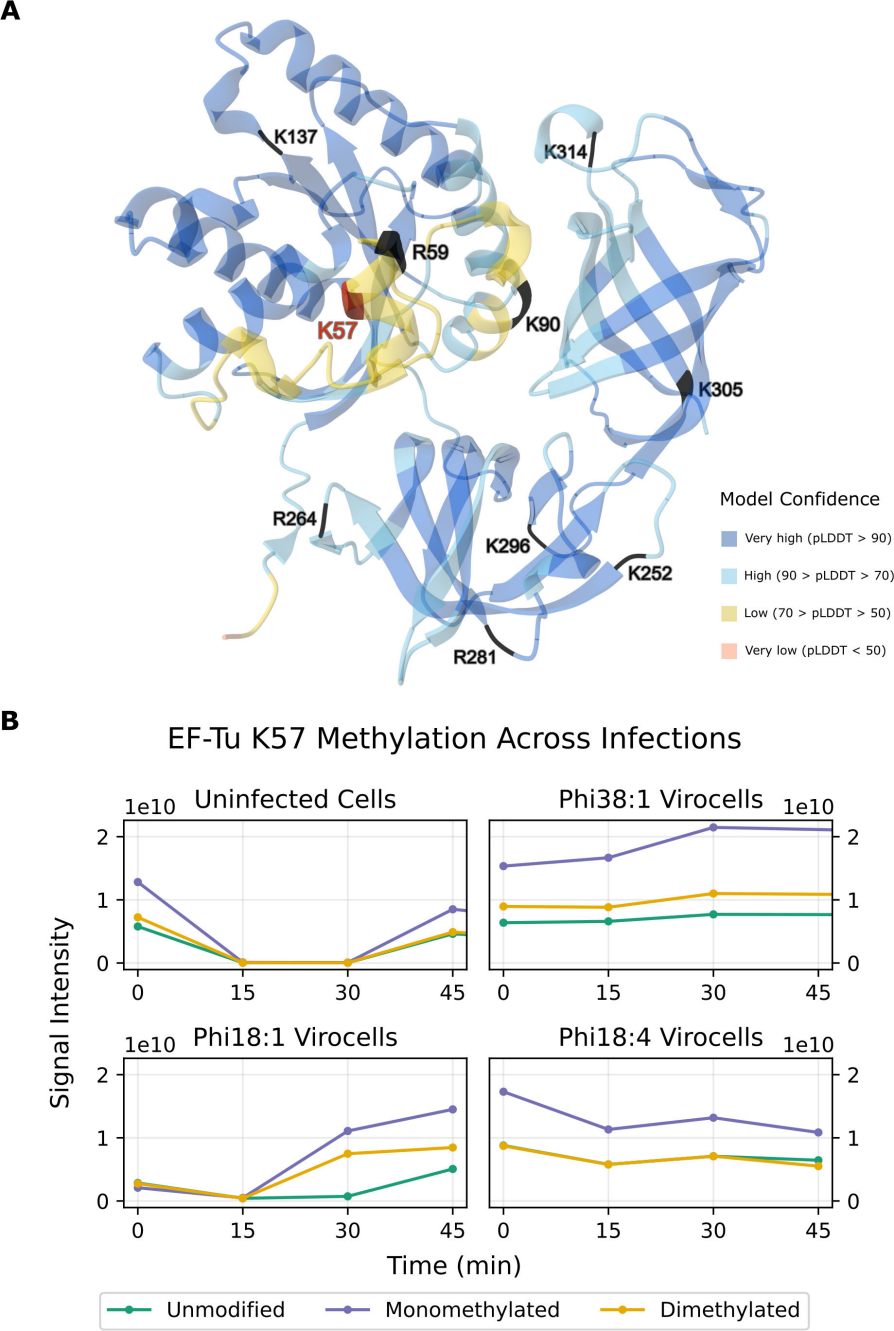
EF-Tu methylation. (**A**) AlphaFold2-predicted structure of EF-Tu in Cba18. Non-labeled residue color indicates pLDDT confidence score. Previously identified methylation events are in maroon; novel methylation identifications are in black. Labeled sites were identified in both Proteome Discoverer and PEAKS. (**B**) Line graphs showing change in EF-Tu peptides’ signal intensities over time for residue K57. Points represent averages of four biological replicates. The three lines correspond to unmodified K57 peptide intensity (green), monomethylated K57 peptide intensity (purple), and dimethylated K57 peptide intensity (yellow).

To do this, we examined the location and prevalence of EF-Tu’s methylation events using a *de novo* sequencing method (PEAKS), in addition to Proteome Discoverer (see Supplemental text). Ten methylation sites were identified by both Proteome Discoverer and PEAKS, and only one of them (K57, corresponding to the K56 in *E. coli* mentioned previously) has been previously identified as methylated in EF-Tu (46, 47, 52). Using an AlphaFold2-predicted structure of the protein (Fig. 4A), we observed that all of the methylated lysines and arginines were predicted to be on the outer surface of the molecule, and many of them occur at the end of beta-strands, which is expected, given the propensity of lysines and arginines to be found there (53). The methylation events were not primarily found in any one domain of the protein but were rather spread spatially throughout the protein (Fig. 4A). Of the 10 residues with identified methylation, K57 was the most readily detected, often accounting for greater than 80% of the summed methylation signal intensity from the 10 residues (Fig. S3). Aligning the amino acid sequence and structure of *E. coli*’s EF-Tu with Cba18’s EF-Tu (Fig. S4) gave a high sequence identity (~69%) and TM-score (~58%), indicating a shared topology (54) between the homologs. Given the similar sequence and structure, the effect of EF-Tu K57 methylation on the protein’s function is expected to be the same as hypothesized for K56 in *E. coli*, where it may slow protein translation due to attenuated GTP hydrolysis.

Because of the prevalence of K57 methylation on EF-Tu and its previous observation as part of a dynamic response to stress (47), we decided to study K57’s methylation as a function of time. Uninfected cells and phi38:1 and phi18:4 virocells all displayed a similar composition of unmodified as well as mono- and dimethylated K57 peptides at T0, with the three peptide forms each having similar, high signal intensities (Fig. 4B; Fig. S5). Phi18:1 virocells, however, had reduced levels of all three peptide forms at the beginning of infection, possibly indicative of another undetected PTM that is occurring at the same lysine or a nearby residue, which would reduce the levels of these unmodified and methylated peptides. However, further investigation of the peptide did not reveal any other prevalent modifications that would have explained this drop-off, so more investigation is needed to determine the source of this decrease in unmodified and methylated peptides. In regard to their high levels of EF-Tu K57 methylation at T0, this may indicate that uninfected cells as well as phi38:1 and phi18:4 virocells all are initially undergoing some growth phase change (46) or stress response (47) as they adjust to their new, diluted environment (Fig. 4B). This response is then reduced in the uninfected cells but continues throughout the infection in the phi38:1 and phi18:4 virocells, perhaps representing the host’s continual stress response to these two phages to slow down translation of viral proteins, a tactic utilized by eukaryotes against oncogenic viruses (55). On the other hand, reduced levels of EF-Tu K57 methylation in early infection for phi18:1 virocells suggest that phi18:1 may be initially inhibiting the stress response of the host cells to prevent a decrease in translation speed: an event that would reduce its ability to produce its proteins and replicate. This strategy of repressing the stress response of the host to aid in infection is known to be used by eukaryotic viruses (56) and proposed to be employed by phi38:1 on a different strain of *C. baltica* it efficiently infects (34). By phi18:1 preventing EF-Tu K57 methylation and thereby stabilizing the translation speed of proteins within the cell, it would improve its ability to replicate compared to phages that did not interfere with this methylation, thereby leading to a larger burst size (B.S.). This is reflected in phi18:1’s larger B.S. (≈90) 2 h post-dilution on Cba18 compared to phi18:4’s (B.S. ≈ 41) (32) and phi38:1’s (undetected after 2 h) (31), though there are a multitude of factors that contribute to phage burst size. Therefore, while EF-Tu seems to be dynamically methylated in response to phage infection, the exact role EF-Tu K57’s methylation plays in phage fitness is unclear and further study will be required to more fully understand this relationship.

These results indicate that K57 on EF-Tu is heavily and dynamically methylated throughout phage infection, that phi38:1 and phi18:4 virocells may continuously activate a stress response in Cba18 cells throughout infection that slows translation of their proteins and decreases burst size, while phi18:1 virocells may employ a strategy of reducing the stress response of the cells to prevent EF-Tu K57 methylation and promote translation of its own proteins.

### Several methylation events on proteins important to phage infection are exclusive to virocells

**Fig 5:**
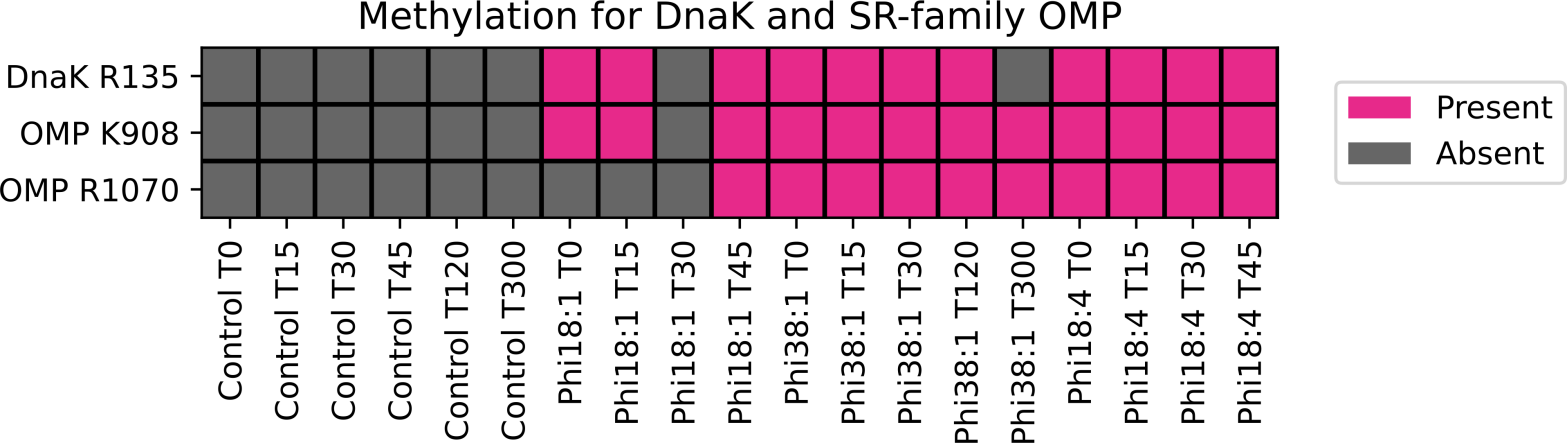
Presence/absence matrix for R135 of DnaK and K908 and R1070 for TonB-linked SusC/RagA family (SR-family) OMP. Presence of methylation on a residue is shown in pink, and absence is shown in gray. Sample names beginning with “control” denote uninfected cells, and sample names beginning with “phi” denote virocells of respective phages.

In order to more fully explore the role of protein methylation in virocells, we expanded our consideration to other possible contributors to phage infection efficiency by examining methylation events that were exclusive to the efficient phage infections (phi18:1 and phi18:4) or the inefficient phage infection (phi38:1). We found no methylation events that were prevalent in the efficient phage virocells relative to the inefficient phage virocells or vice versa. However, we also searched for potential general host responses to phage infection by examining methylation events that were only found in all three virocells and not in uninfected cells. Since there were many methylation events that fulfilled this criterion (Fig. 3C, *n* = 95), these were further filtered down by focusing on methylation events with the highest prevalence that occurred on proteins of known/suspected importance to phage infection. Two proteins that had prevalent and unique-to-virocell methylation events were the molecular chaperone DnaK on arginine (R) 135 and a TonB-linked outer membrane protein (OMP) in the SusC/RagA family (hereafter referred to as “SR-family OMP”) on K908 and R1070 (Fig. 5). Dimethylated R135 on DnaK was detected in every virocell time point except for T30 in phi18:1 virocells and T300 in phi38:1 virocells. For the SR-family OMP, dimethylated K908 was detected in all virocell time points except for T30 in phi18:1 virocells, and dimethylated R1070 was detected at T45 for phi18:1 virocells and at every time point for 38:1 and phi18:4 virocells (Fig. 5). These phage-specific methylation events were not found to be a result of protein abundance changes, as the proteins maintained similar or even slightly higher levels in the uninfected condition compared to the infected conditions (Fig. S6), suggesting that DnaK and the SR-family OMP undergo specific methylation events exclusive to virocells. Additionally, the lack of these methylation events at T30 in phi18:1 virocells aligns with the observed widespread reduction in methylation events occurring at this time point (Table 1), though the source of this reduction is unknown.

Importantly, both of these proteins have known or suspected interactions with phage infection. DnaK has been previously found to be required for DNA replication of the *E. coli* phage lambda (57) and has been seen in *E. coli* to be targeted by the phage M13 with another common PTM, phosphorylation (58). The dimethylated residue R135 in DnaK is found within a predicted beta sheet in the N-terminal ATPase domain of the protein (Fig. S7). Dimethylating this residue may disrupt hydrogen bonds within the beta sheet, influencing the overall structure of the protein and its ability to bind to ADP/ATP. This, in turn, may modify the behavior of the protein to a function that better suits the needs of the phage for replication. For the SR-family OMP, K908 and R1070 are each likely found on the extracellular-facing side of the protein (Fig. S8). As OMPs are often targets of phage adhesion to host cells (59–61), methylation of these residues may serve to prevent phage adhesion. If this is the case, either the host or the phage could be inducing their methylation: the host, if it detects phage in the environment and wants to prevent adsorption (19), and the phage, if it has already infected the cell and wants to prevent other phages from adsorbing, a strategy previously observed in the *E. coli* phage T5 (62).

All in all, given the virocell-exclusive methylation patterns seen in them and their known relationship to phage infections, there is strong evidence that the functions of DnaK and the SR-family OMP are being modified in response to phage infection through methylation, though whether this is phage or host driven is unknown and requires further investigation.

### Gliding motility proteins, important for phage adsorption, have extensive methylation signatures

Inspection of the proteome data sets also revealed a notable number of methylation events (*n* = 23) in Cba18’s gliding motility proteins (Table 4). In bacteria, protein methylation has been observed to be crucial for regulating the activity of key proteins involved in gliding motility, controlling the direction and speed of cell movement by influencing the mechanism that connects the cell to the substrate surface (25). Additionally, gliding motility proteins can be used for initial phage adsorption and cell entry, and mutations in these genes lead to extracellular phage resistance in Cba18 and other related Flavobacteria (10, 63, 64). This suggests that gliding motility protein methylation could be another mechanism by which the bacterial host controls phage infection.

**TABLE 4:** Methylation events on gliding motility proteins^*a*^

Gene accession	Protein	Location and number of methyl groups
Ga0325142_112129	GldB	R105(D)
Ga0325142_11767	GldJ	R154(D), R189(D), K198(D), R255(D), K307(D), K412(D)
Ga0325142_11893	GldK	K201(D), R301(D), K455(D)
Ga0325142_11892	GldL	K101(D), K108(D), R145(D), K190(D)
Ga0325142_11891	GldM	K134(D), K154(D), K207(D), K239(D), K247(D), R344(D), K404(D)
Ga0325142_11890	GldN	K102(D), R193(D)

^*a*^ Identified methylation state for a lysine (K) or arginine (R) residue is denoted by “(M),” “(D),” or “(T),” for monomethylation, dimethylation, and trimethylation, respectively. All methylation events were automatically identified by Proteome Discoverer and manually reevaluated by inspection of MS2 spectra.

Of the 11 detected gliding motility proteins, 6 (GldB, GldJ, GldK, GldL, GldM, and GldN) had evidence of methylation, including all 4 Gld proteins that form the core of the type IX secretion system (GldK, GldL, GldM, and GldN) (65). The type IX secretion system is unique to the Bacteroidota phylum (65) and is involved in the secretion of proteins onto the extracellular-facing side of the outer membrane, including two adhesins (SprB and RemA) crucial for gliding motility that may serve as phage receptors (63, 66). There did not appear to be any temporal or phage-related change in the gliding motility proteins, as most methylation events were infrequent, but a few locations of the methylation events did stand out. On GldM, a missense mutation of G304R in the second domain has been seen to confer phage resistance (63), indicating that this domain most likely plays an important role in the folding or function of GldM exploited by the phage for infection. Two of the methylation events in GldM are predicted to occur in this domain, namely, dimethylation of K239 and K247. While neither of these residues are predicted to be spatially near the mutated residue (>20 angstroms) (Fig. S9), they are in a similar spatial region as the mutation site, suggesting that a structural change potentially induced by their methylation may have a similar effect on the protein’s function. GldJ contains a predicted formylglycine-generating enzyme (FGE) sulfatase domain, which is responsible for activating sulfatases within the cell. Half of the methylated residues on GldJ occur within predicted alpha helices or beta sheets (Fig. S10A), and all of the methylation sites in this protein lie within or close to this predicted FGE sulfatase domain (Fig. S10B), suggesting a possible role of methylation in modifying the functioning of GldJ. In summary, gliding motility proteins, known to be crucial for some phages for infection, are frequent targets of methylation that may impact their roles in uninfected as well as infected cells.

### Conclusions

The dynamic interaction between bacteria and their phages is a prevalent and highly evolved process. PTMs can be strategically utilized by both the host and the attacking phage to gain survival advantages. Altogether, these results indicate that protein methylation, like other PTMs in bacterium-phage interactions and methylation in eukaryotic viruses, serves as an additional mode of protein regulation during phage infection. Our data reveal significantly elevated levels of host methylpeptides in virocells, even in early infection. We report for the first time phage protein methylation during infection, some of which is maintained within the free virion form of the phage. We also highlight several methylation events that may play important roles in infection. Specifically, EF-Tu is observed to be differentially methylated over time between infected and uninfected cells, and both DnaK and a SusC/RagA family OMP exhibit infection-specific methylation events. Additionally, gliding motility proteins, known to play a critical role in phage resistance mechanisms in Cba18, display numerous methylation events that likely impact their function. In total, our results provide the first global view of methylation dynamics across both host and phage proteins during infection, as well as identify methylation events on specific proteins that represent promising targets for future mechanistic investigation of their role in phage infection. This, along with future work, will serve to fill in missing knowledge as we seek to understand the intricacies of how phages attack and metabolically reprogram bacteria and how bacteria attempt to defend themselves.

## MATERIALS AND METHODS

### Strains, growth conditions, and time-resolved sampling

Bacterial and viral strains (Fig. 1A; Table S5), growth conditions, and sampling methods for the virocell and uninfected control samples are detailed elsewhere (S. Rajakaruna, C. Howard-Varona, M. Urvoy, R. AminiTabrizi, C. Ayala-Ortiz, M. Gittrich, N. Solonenko, M. Burris, C. Sanderson, C. Noel, J. Leopold, L. Quillin, K. Doshi, L. Walker, M. Sullivan, and M. Tfaily, submitted for publication). Briefly, cells were grown in biological quadruplicates; phages were added and allowed to adsorb; and samples were diluted to synchronize phage infections. Samples were then collected at their respective time points post-dilution (Fig. 1B). An expanded description is provided in the Supplemental text.

### Protein extraction and liquid chromatography tandem mass spectrometry measurement

Protein extraction and liquid chromatography tandem mass spectrometry (LC-MS/MS) analysis was carried out as detailed in the Supplemental text. Briefly, cells were lysed; proteins were denatured using heat; and the PAC method (67) was used with magnetic beads to isolate proteins from samples. Proteins were then trypsin digested into peptides, which were collected for LC-MS/MS analysis. Peptides were analyzed by LC-MS/MS using a Vanquish UHPLC coupled to a QExactive Plus mass spectrometer (Thermo Fisher Scientific) with data-dependent acquisition.

### Peptide identification and data analysis

The majority of peptide identification was done using Proteome Discoverer version 2.5 (Thermo Fisher Scientific), with further details and parameters provided in the Supplemental text. Additionally, information regarding other software and online tools used, as well as statistical tests, is also given in the Supplemental text.

## Supplementary Material

Reviewer comments

## Data Availability

All raw data generated by mass spectrometry can be found at the MassIVE repository under data set identifier MSV000098485 and at ProteomeXchange under data set identifier PXD066074.

## SUPPLEMENTAL MATERIAL

Supplemental figures msystems.00012-26-s0001.pdf Figures S1 to S10.

Supplemental text msystems.00012-26-s0002.docx Additional experimental details.

Supplemental tables msystems.00012-26-s0003.xlsx Tables S1 to S5

OPEN PEER REVIEW reviewer-comments.pdf An accounting of the reviewer comments and feedback.

ASM does not own the copyrights to Supplemental Material that may be linked to, or accessed through, an article. The authors have granted ASM a non-exclusive, world-wide license to publish the Supplemental Material files. Please contact the corresponding author directly for reuse.
