# Supplementary material for: Detection and characterization of protein methylation in bacteriophages and their host, Cellulophaga baltica, during infection
Source: mSystems. 2026 May 18;11(6):e00012-26. doi: 10.1128/msystems.00012-26 (PMC13288928; doi:10.1128/msystems.00012-26)
Supplement: Supplemental figures — Figures S1 to S10. [file msystems.00012-26-s0001.pdf]

Detection and Characterization of Protein Methylation in Bacteriophages and Their Host, *Cellulophaga baltica,* During Infection

Andrew J. Stai[1,2], Cristina Howard-Varona[3,4], Marion Urvoy[3,4], Marissa R. Gittrich[3,4], Matthew B. Sullivan*[3,4,5], Robert L. Hettich*[1]

[1] Biosciences Division, Oak Ridge National Laboratory, Oak Ridge, TN 37830

[2] The Bredesen Center for Interdisciplinary Research and Graduate Education, University of Tennessee, Knoxville, TN 37996

[3] Department of Microbiology, The Ohio State University, Columbus, OH 43210

[4] Center of Microbiome Science, The Ohio State University, Columbus, OH 43210

[5] Department of Civil, Environmental and Geodetic Engineering, The Ohio State University, Columbus, OH 43210

*Co-Correspondence: Robert Hettich, Oak Ridge National Lab, Oak Ridge, TN 37830, hettichrl@ornl.gov, 865-241-6373

Matthew Sullivan, The Ohio State University, Columbus, OH 43210, sullivan.948@osu.edu, 614-247-1616

Notice: This manuscript has been authored by UT-Battelle, LLC under Contract No. DE-AC05-00OR22725 with the U.S. Department of Energy.

SUPPLEMENTARY FIGURES

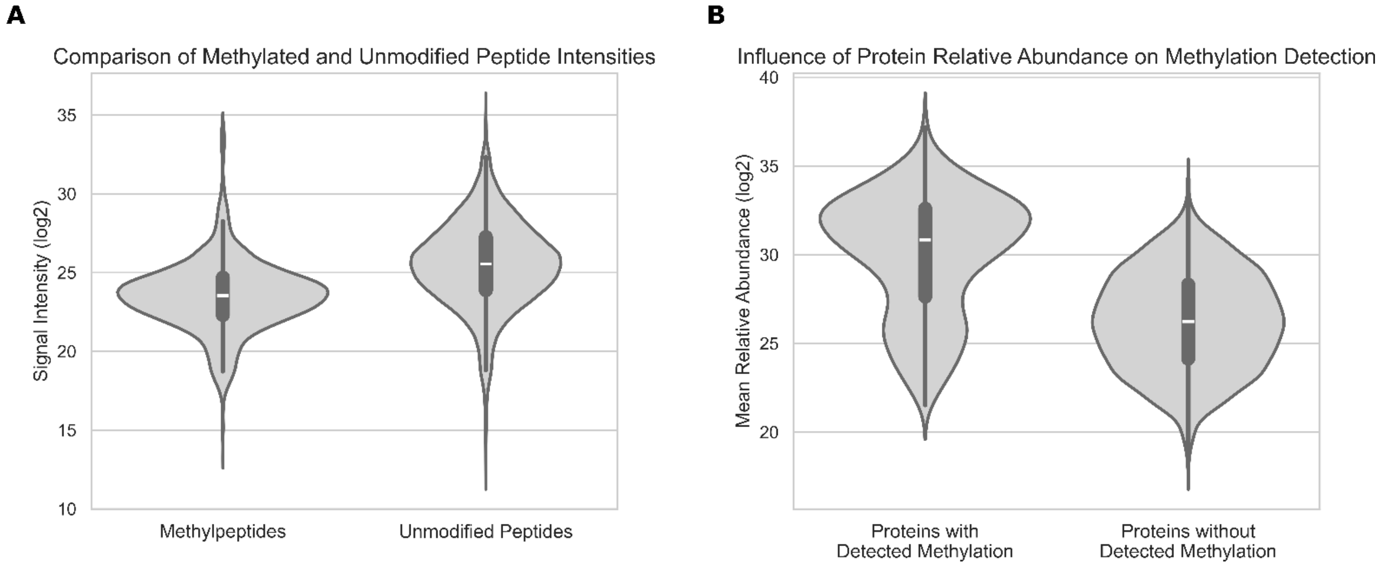

Supplementary Figure 1: A) Violin plot comparing signal intensities of peaks from methylpeptides (n = 17,313) with peaks from unmodified peptides (n = 1,061,187). B) Violin plot showing relationship between protein relative abundance and methylation detection. Left violin displays distribution of mean relative abundances of proteins containing at least one peptide with detected methylation (n = 552). Right violin displays distribution of mean relative abundances of proteins without any peptide with detected methylation (n = 2,927). A-B) A small boxplot is shown within the violins with medians (white) as well as first and third quartiles shown (thick dark grey). Boxplot lines beyond first and third quartiles represent a distance 1.5 times the interquartile range.

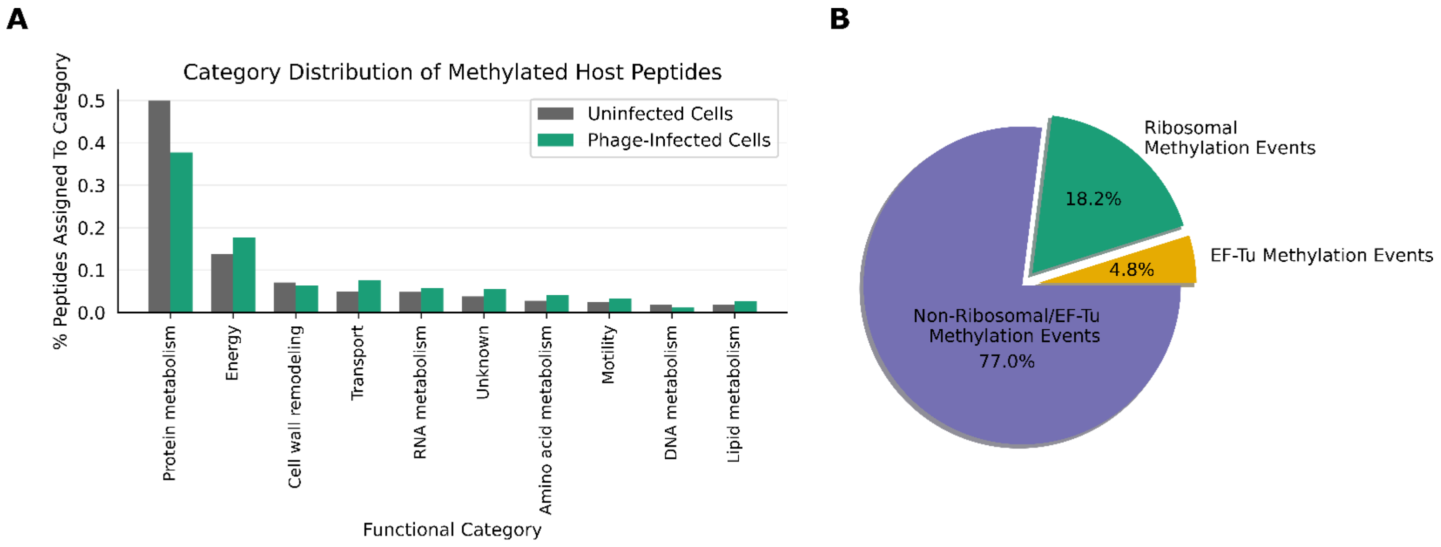

Supplementary Figure 2: A) Paired barchart showing distribution of methylated peptides coming from control (grey) and virocell (green) conditions among ten most common manually curated protein functional categories. B) Pie chart showing percentage of methylation events that were found on ribosomes (green), EF-Tu (yellow), or other host proteins (purple).

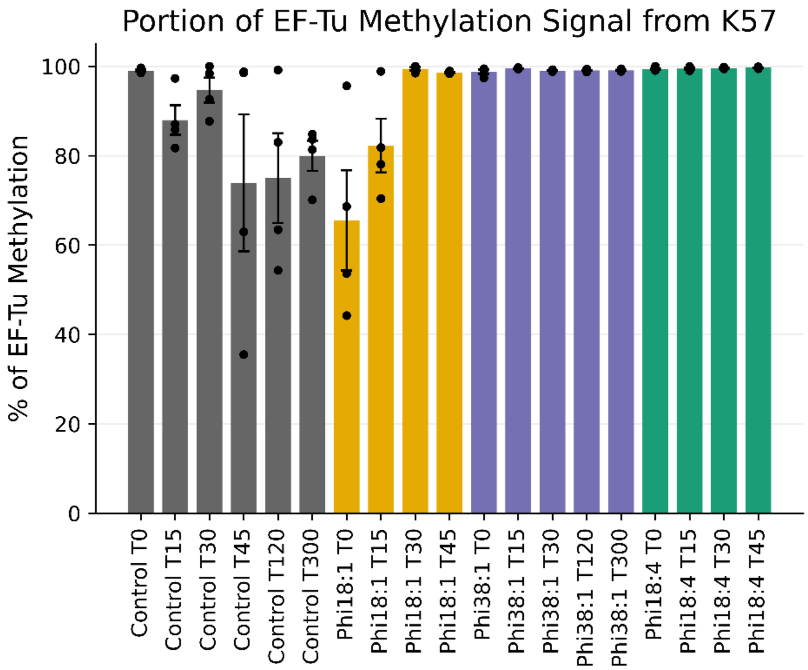

Supplementary Figure 3: Barchart showing portion of EF-Tu's total methylation signal coming from K57 methylated peptides. Bar heights represent averages of biological replicates ($n = 4$) with replicates plotted in black. SEM error bars are shown with vertical brackets. Sample names beginning with "Control" denote uninfected cells, and sample names beginning with "Phi" denote virocells of respective phages.

A

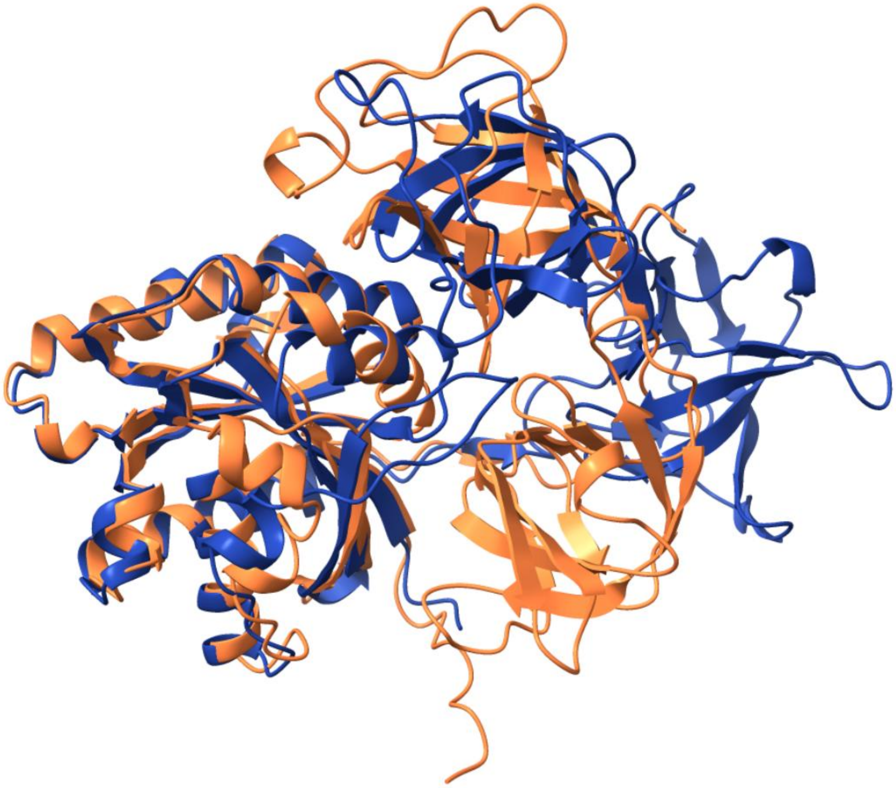

B

C. baltica 18   39 … G L S E L R S F D S I D N A P E E K E R G I T I N T S H V E Y S T A N … 75

E. coli K12   39 … Y G G A A R A F D Q I D N A P E E K A R G I T I N T S H V E Y D T P T … 75

Supplementary Figure 4: Cba18's EF-Tu similarity with *E. coli's* EF-Tu. A) AlphaFold2-predicted structure of EF-Tu from Cba18 compared to x-ray crystallography structure from *E. coli* (PDB ID: 1EFC). The AlphaFold2-predicted structure is shown in orange, and the x-ray crystallography of *E. coli*'s EF-Tu is shown in blue. The RMSD was 4.71 and TM-score was 0.58418, with both calculated by US-align (53). B) Amino acid sequences surrounding K57 (residues 40-74 shown) of EF-Tu from Cba18 and *E. coli* K-12, with identical residues highlighted in green, differing residues highlighted in grey, and K57 surrounded by a box for clarity.

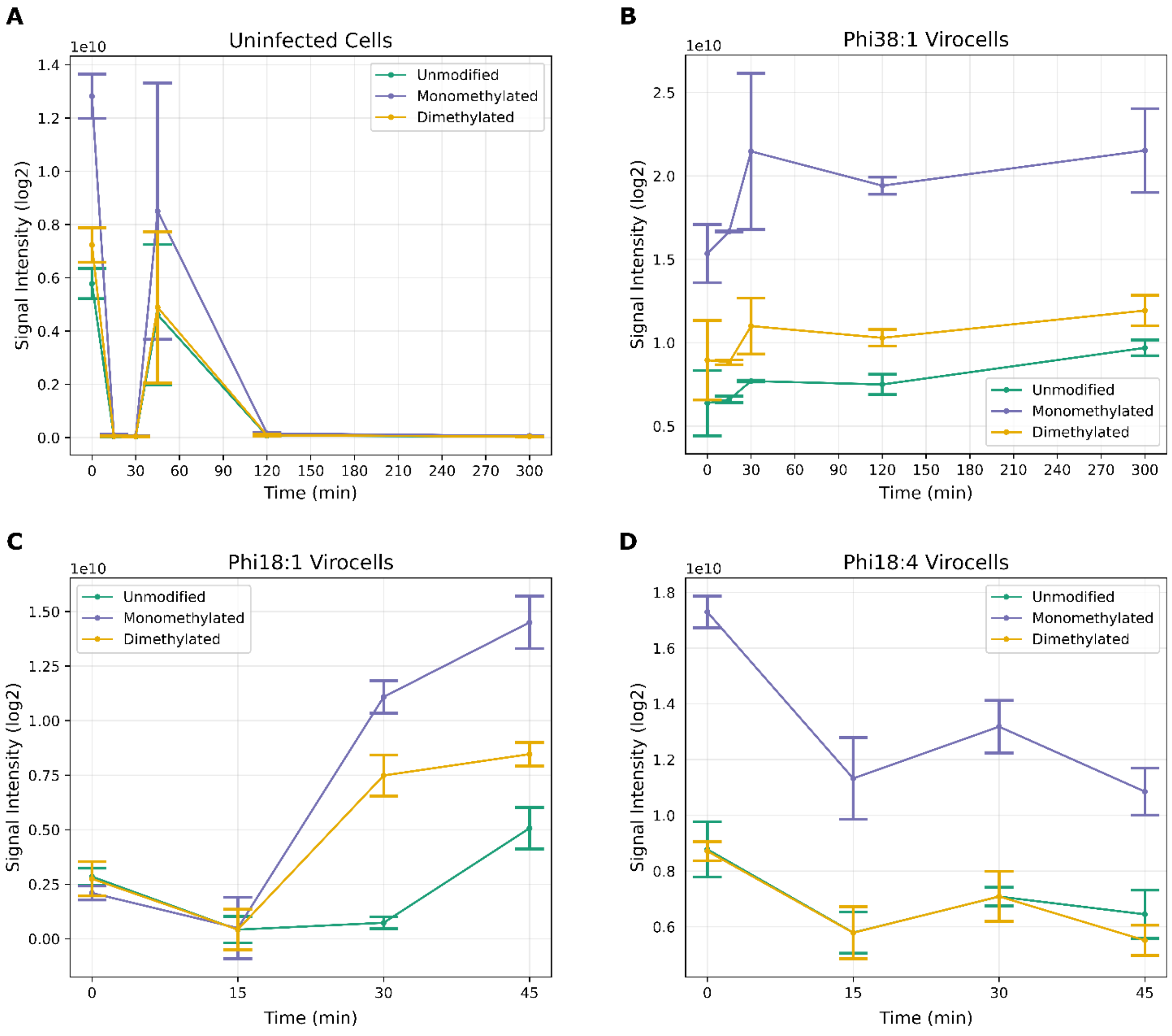

Supplementary Figure 5: Expanded view of Figure 4B. Line graphs showing change in EF-Tu peptides' signal intensities over time for residue K57 for uninfected cell (A), phi38:1 virocells (B), phi18:1 virocells (C), and phi18:4 virocells (D). The three lines correspond to: unmodified K57 peptide intensity (green), monomethylated K57 peptide intensity (purple), and dimethylated K57 peptide intensity (yellow). Points represent averages of four biological replicates. SEM error bars are shown with vertical brackets and are colored to match their respective peptide form.

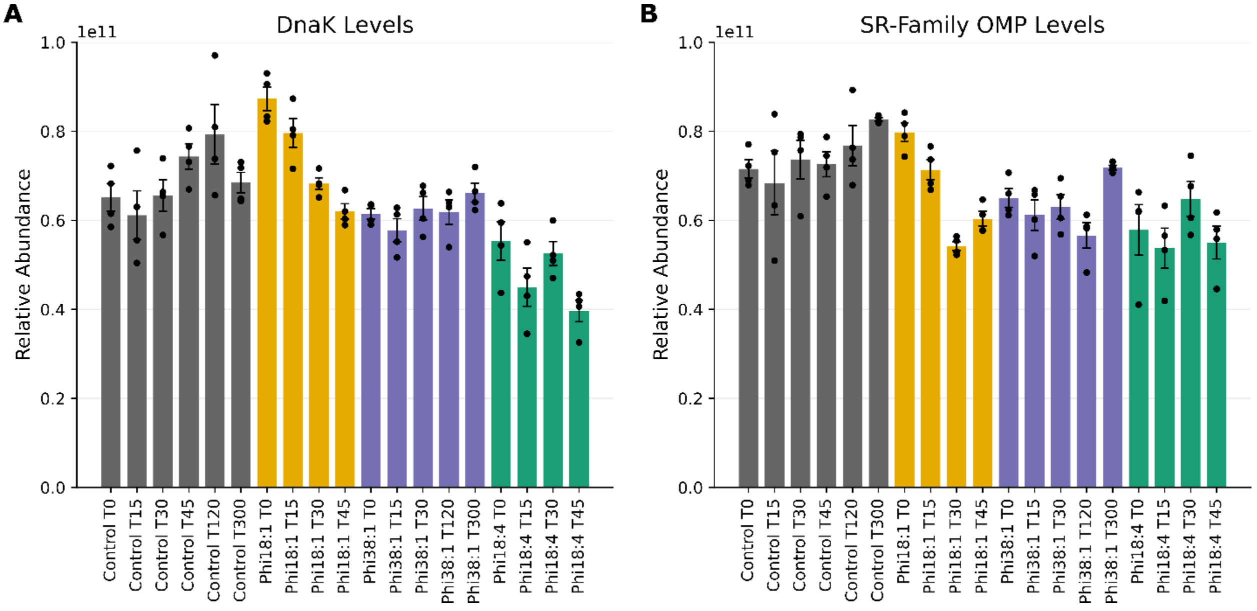

Supplementary Figure 6: Barcharts showing relative abundances of (A) DnaK and (B) SusC/RagA Family OMP (SR-Family OMP) in all samples. Bar heights were calculated by averaging peptide intensities across biological replicates ($n = 4$) and summing all peptide intensities that belonged to each protein at that time point. Individual biological replicate values are plotted in black. SEM error bars are shown with black vertical brackets. Sample names beginning with “Control” denote uninfected cells, and sample names beginning with “Phi” denote virocells of respective phages.

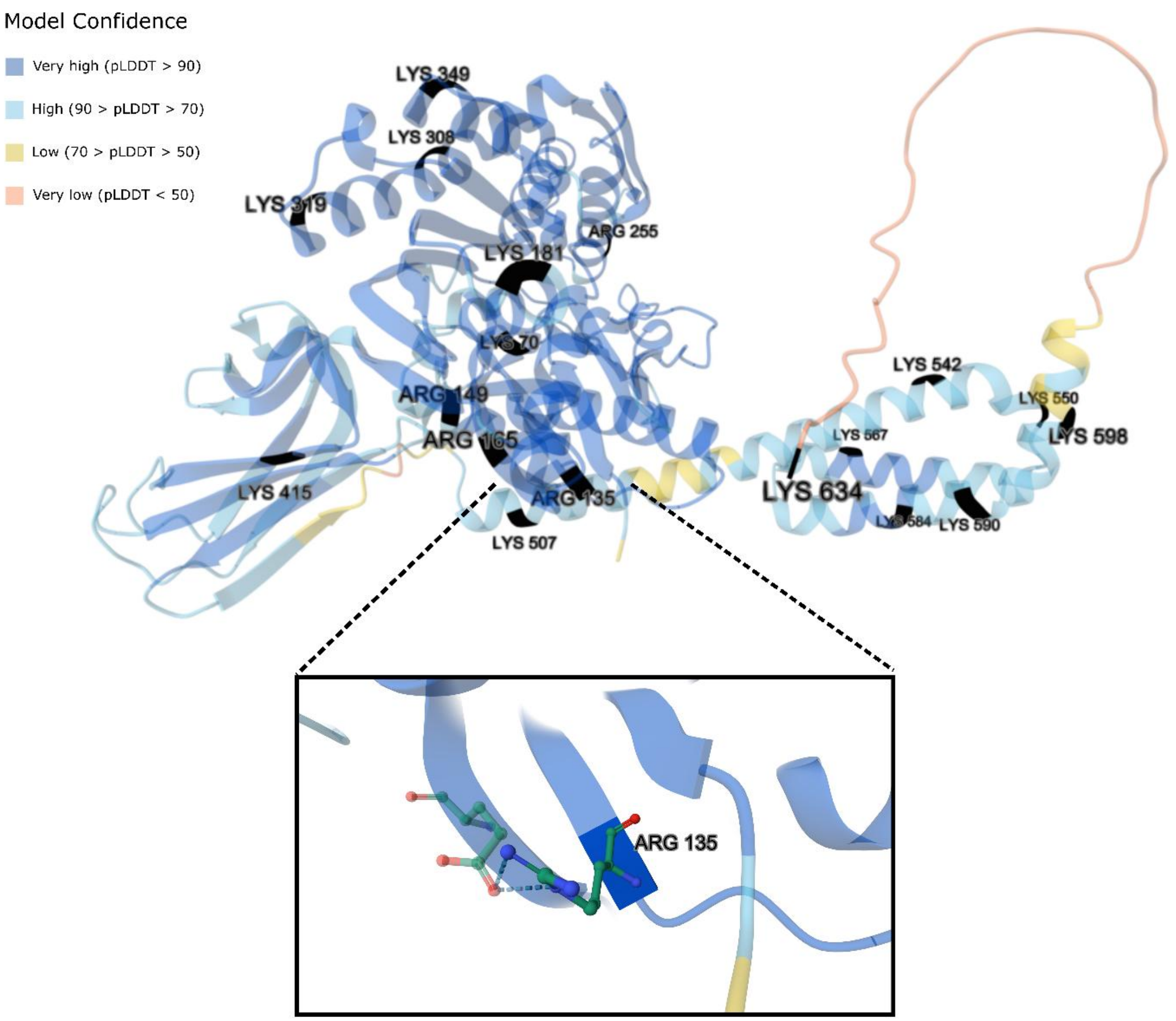

Supplementary Figure 7: AlphaFold2-predicted structure of Cba18's DnaK with detected methylated residues labeled and larger view of R135 with ball and stick representation and beta sheet hydrogen bonds represented as blue dashed lines. Residue coloring based on plDDT confidence scores.

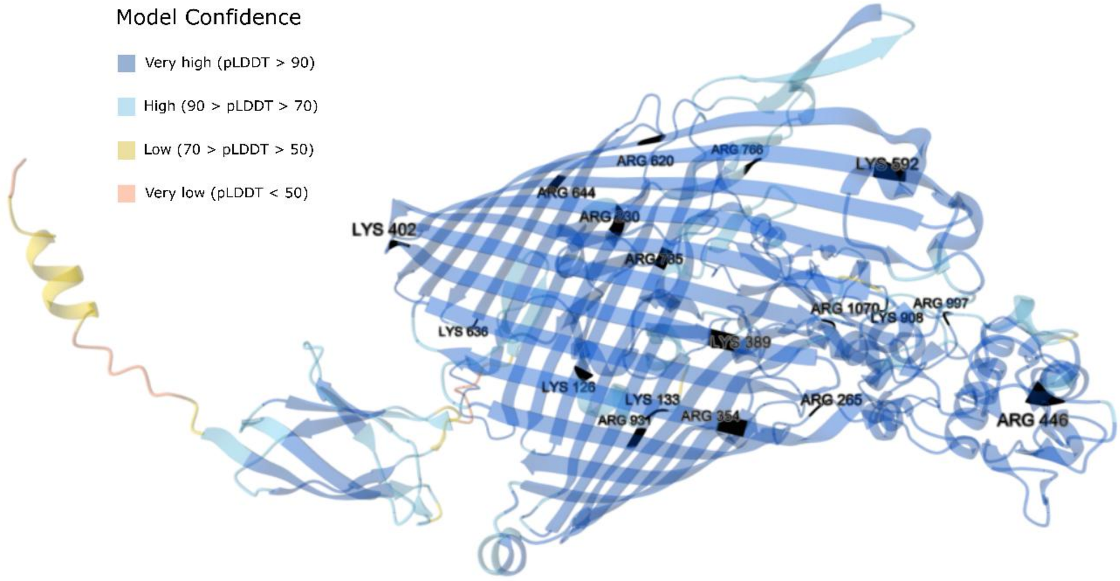

Supplementary Figure 8: AlphaFold2-predicted structure of Cba18's TonB-linked OMP in SusC/RagA family with detected methylated residues labeled. Residue coloring based on plDDT confidence scores.

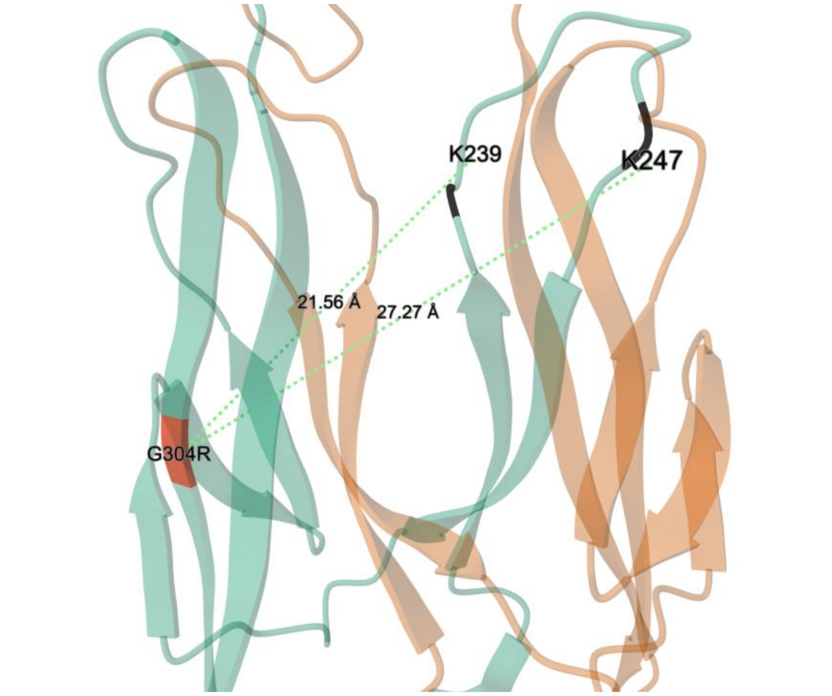

Supplementary Figure 9: Crystal structure of the second domain of *Flavobacterium johnsoniae*'s gliding motility protein M (PDB ID: 6EY4), a homolog of Cba18's GldM with a shared topology (TM-score: 56%; calculated by US-align (53)). Green and orange colors correspond to chain ID. Red-colored G304 residue corresponds with mutated (glycine-to-arginine) residue seen to confer phage resistance. Detected methylated residues are shown in black. Distances in angstroms between mutated and methylated residues are shown on green dotted lines.

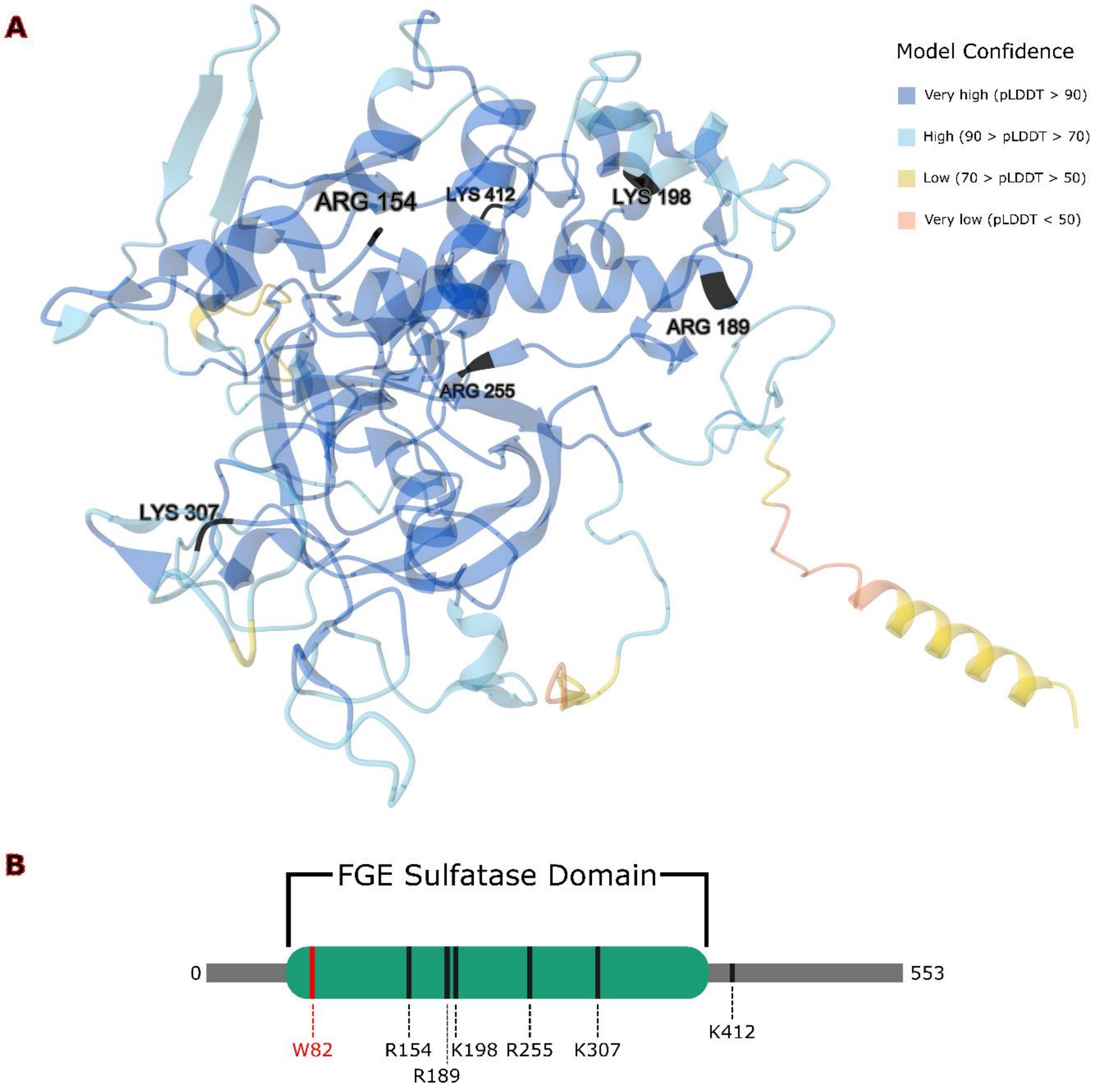

Supplementary Figure 10: Methylation of Cba18's Gliding Motility Protein J. A) AlphaFold2-predicted structure with detected methylated residues labeled. Residue coloring based on plDDT confidence scores. B) Location of mutated (red) and methylated (black) residues in primary sequence and in respect to InterPro-predicted FGE sulfatase domain (Pfam ID PF03781, green).
