# Supplementary material for: Detection and characterization of protein methylation in bacteriophages and their host, Cellulophaga baltica, during infection
Source: mSystems. 2026 May 18;11(6):e00012-26. doi: 10.1128/msystems.00012-26 (PMC13288928; doi:10.1128/msystems.00012-26)
Supplement: Supplemental text — Additional experimental details. [file msystems.00012-26-s0002.docx]

**Detection and Characterization of Protein Methylation in Bacteriophages**

**and Their Host, *Cellulophaga baltica,* During Infection**

Andrew J. Stai^1,2^, Cristina Howard-Varona^3,4^, Marion Urvoy^3,4^, Marissa R. Gittrich^3,4^,

Matthew B. Sullivan*^3,4,5^, Robert L. Hettich*^1^

^1^ Biosciences Division, Oak Ridge National Laboratory, Oak Ridge, TN 37830

^2^ The Bredesen Center for Interdisciplinary Research and Graduate Education, University of Tennessee, Knoxville, TN 37996

^3^ Department of Microbiology, The Ohio State University, Columbus, OH 43210

^4^ Center of Microbiome Science, The Ohio State University, Columbus, OH 43210

^5^ Department of Civil, Environmental and Geodetic Engineering, The Ohio State University, Columbus, OH 43210

*Co-Correspondence: Robert Hettich Matthew Sullivan

Oak Ridge National Lab, The Ohio State University

Oak Ridge, TN 37830 Columbus, OH 43210

hettichrl@ornl.gov sullivan.948@osu.edu

865-241-6373 614-247-1616

Notice: This manuscript has been authored by UT-Battelle, LLC under Contract No. DE-AC05-00OR22725 with the U.S. Department of Energy. The United States Government retains and the publisher, by accepting the article for publication, acknowledges that the United States Government retains a non-exclusive, paid-up, irrevocable, world-wide license to publish or reproduce the published form of this manuscript, or allow others to do so, for United States Government purposes. The Department of Energy will provide public access to these results of federally sponsored research in accordance with the DOE Public Access Plan (<http://energy.gov/downloads/doe-public-access-plan>).

**SUPPLEMENTAL TEXT**

Strains, growth conditions, and time-resolved sampling

Cells were grown in marine LB (MLB), phages were added to cells and allowed to adsorb for 10-15 minutes, and then samples were diluted ten-fold to synchronize the infection. Two 35 mL samples of each biological replicate were then taken at their respective time points post-dilution (T0 being immediately after dilution, T15 being 15 minutes after dilution, etc.) (Figure 1B). The two 35 mL samples for each biological replicate were centrifuged, and subsequent cell pellets were resuspended in phosphate buffered saline (PBS), combined with the other sample from the same biological replicate, centrifuged a second time, resuspended in 1 mL PBS, centrifuged a third time, flash frozen after pouring off the supernatant, and stored at -80°C. All centrifugations were done at 10,000 x g at 4°C for 10 minutes for the 35 mL samples and 5 minutes for the 1 mL samples.

For proteomics on the concentrated free virions, phages were amplified on their preferred hosts (Cba18 for phi18:1 and phi18:4; *Cellulophaga baltica* strain #38 for phi38:1) using a standard plaque assay method as described before (1).

Protein extraction and liquid chromatography tandem mass spectrometry measurement (LC-MS/MS)

Cell pellets were resuspended in 100 mM ammonium bicarbonate (ABC100) and lysed through bead beating with 0.15 mm zirconium oxide beads. After addition of 20% sodium dodecyl sulfate (SDS) and dithiothreitol (DTT) to samples to achieve 4% SDS and 10mM DTT, proteins were denatured by heating at 90℃ for 10 minutes, centrifuged, and alkylated by incubation with 30mM iodoacetamide. The PAC method (2) was used to isolate proteins from samples by adding 300 μg of hydrophobic magnetic beads (SpeedBead Magnetic Carboxylate; GE Healthcare UK) and acetonitrile (ACN) to a concentration of 80% ACN. After room temperature incubation for 20 minutes, samples were placed on a magnetic rack, and the supernatant was removed. Beads were then washed with ACN, which was aspirated off, followed by rewashing the beads with 70% ethanol and aspirating off the ethanol. Proteins on beads were resuspended in 4% SDS, heated for 10 minutes at 90℃, and an aliquot was used for protein quantification using the Scopes method of a NanoDrop OneC (Thermo Fisher Scientific) at 205 nm. The PAC method on the unaliquoted portion was then repeated after the bead addition step through the 70% ethanol wash and aspiration. Beads were then resuspended in 200 μL of ABC100. and proteins were trypsin-digested into peptides overnight with a 1:75 trypsin weight:protein weight ratio at 37℃. Trypsin was then added again for three hours for a second digestion while shaking at 600 rpm. Peptides were then filtered using a 10 kDa MWCO filter plate (AcroPrep Advance, Omega 10 K MWCO) and quantified again using a NanoDrop OneC.

For each sample, 3 μg of peptides were loaded onto a liquid chromatography tandem mass spectrometry (LC-MS/MS) setup using a Vanquish ultra-HPLC system coupled with a QExactive-Plus mass spectrometer (Thermo Fisher Scientific) (Figure 1C). An in-house 100 μm inner diameter Kinetex C18 reverse-phase column was used to separate peptides by hydrophobicity. The LC solvent gradient was as follows: 100% solvent A (98% water, 2% ACN, 0.1% formic acid) from 0 to 30 minutes; a linear gradient of solvent B (70% ACN, 30% water, 0.1% formic acid) from 0 to 30% from minutes 30 through 220; a linear gradient of 0 to 100% solvent B from 220 to 255 minutes; followed by 100% solvent A until 275 minutes. Peptides were analyzed in positive mode using data-dependent acquisition with the top 20 abundant peaks fragmented for further identification. The following settings were used: 400-1500 m/z mass range, 1.8 m/z isolation window, 70 K MS resolution, 17.5 K MS/MS resolution, and a normalized collisional energy of 27 for fragmentation. Charge states of +1 and +6-8 were excluded.

Peptide identification and data analysis

As peptides and not whole proteins were measured by mass spectrometry, any analysis in this work involving quantification of methylation was done on the peptide level. Peptide identification and quantification was done automatically by Proteome Discoverer version 2.5 (Thermo Fisher Scientific) by searching against a database containing a FASTA file with genomes of *Cellulophaga baltica* strain 18, phages phi18:1, phi18:4, and phi38:1, as well as a FASTA file containing common contaminants. Searching was done using the Sequest HT algorithm and peptide validation was done by Percolator. The target global FDR was set to 1%, the max number of missed cleavages set to 2, and the minimum peptide length set to 6. The precursor mass tolerance was set to 2 parts-per-million, and the fragment mass tolerance was set to 0.02 daltons. Cysteine carbidomethylation was set as a static modification and methionine oxidation, lysine/arginine (KR) monomethylation, KR dimethylation, and trimethylation were set as dynamic modifications. Peptide intensity values were median centered in python using a custom script available at <https://github.com/andrewstai/Virocell-Methylation-Paper>. All median-centered peptide intensity values for each sample can be found at the study’s MassIVE repository under dataset identifier MSV000098485 under the filename “Median_centered_peptide_intensities.csv”.

“Methylation event,” a term used throughout the paper for brevity, is here defined as a detected mono-, di-, or trimethylation of a residue on a protein (e.g., dimethylation of lysine 100 on protein X). If the same residue is detected as having multiple methylation states (e.g., lysine 100 on protein X is detected as both mono- and dimethylated), then all methylation states of that residue are treated as separate methylation events. For other relevant definitions, see Supplementary Table 3.

Due to (i) a relatively low number of methylated peptides (methylpeptides) being identified compared to unmodified peptides (Table 1) and (ii) methylpeptides being on average lower abundance than unmodified peptides (Supplementary Figure 1A), which would reduce the intensity of their corresponding fragmentation peaks in respect to other co-isolated peptides and thereby reduce the ability to confidently identify methylpeptides in complex spectra, we suspected the false discovery rate (FDR) of methylpeptides would be higher than the target 1% FDR for all peptides. As a result, all methylation events specifically named in this work were manually validated by inspection of MS2 fragmentation spectra in Proteome Discoverer, resulting in visual inspection of ~190 methylpeptides in total. Spectra complexity, retention time shift, peak intensities, and proportion of peptide sequence covered by *b* and *y* ions all were used in manual validation. Additionally, if unmodified versions of the peptide were detected, the relative intensity of the peaks of the suspected methylated peptide were compared to the relative intensity of the peaks of the unmethylated peptide of the same charge state. Methylpeptides were considered true positives if: (i) they fell within a range of retention time shifts expected of the addition of one or more methyl groups, (ii) the majority of the peptide’s residues had representative gaps between peaks corresponding to their mass, (iii) the spectra was either primarily composed of peaks that matched the peptide sequence or had explainable non-matching peaks, (iv) the relative intensity of the peaks in the methylated peptide matched the relative intensity of the peaks in the unmethylated peptide of the same charge state, if available. Of the methylpeptides that did not pass validation, a minority could be identified as false positives. Rather, the majority of these had insufficient support for their identification but were still not included in the final analysis.

Although mass spectrometry is well-suited for identifying methylated peptides, correct localization of the methylation event on the peptide can still be a challenge if there is a lack of peaks in the fragment spectrum that cover the anticipated location of the methylation event. Due to trypsin’s cut sites primarily occurring on the C-terminal side of lysines and arginines and our automated searches for methylation events being restricted to arginines and lysines, many methylation events reported were located at the C-terminal end of peptides. While peptides tend to fragment better in the middle of their sequence, the majority of the time there were still *y*_1_ *or y*_2_ ions with the correct mass shift present in the spectra of methylpeptides, providing evidence for localization of the methylation event down to, at the very least, the last two residues of the peptide. However, especially when *y*_1_ ion fragment peaks were missing, there was a potential for ambiguity in correct site localization of the methylation event.

Further investigation of methylation of Elongation Factor Tu was done by PEAKS Xpro 2020 (Bioinformatics Solution Inc.). A sequence database search was performed using default parameters with carbidomethylation as a fixed PTM and methionine oxidation, KR monomethylation, KR dimethylation, and trimethylation as variable PTMs.

Proteins amino acid sequences were aligned using NCBI’s protein-protein BLAST (3). Protein structures were predicted with default parameters by ColabFold v1.5.5 (4), which utilizes AlphaFold2 (5) and AlphaFold2-multimer (6). Labeled protein structures were visualized by Mol* from the Protein Data Bank (PDB), and structural alignment was done by US-Align (7) and visualized by UCSF ChimeraX (8). Tenacibaculum platacis’s lysine demethylase (accession number 0ABM9NWM9) amino acid sequence was taken from UniProt (9). Crystal structures for EF-Tu (PDB ID 1EFC) (10) and GldM (PDB ID 6EY4) (11) were taken from the PDB. Functional domain prediction for GldJ was done using InterProScan (12) with default parameters.

All statistical analysis was done in Python (v3.13.7) primarily using the NumPy (v2.3.3) and Pandas (v2.3.3) packages. All significance values presented in this work came from two-sided, two-sample Mann-Whitney U tests that were done using the SciPy (v1.6.12) Stats library. Standard Error of the Mean (SEM) was calculated for all depicted error bars using the same SciPy Stats library. All reported p-values were corrected for multiple testing as Benjamini-Hochberg adjusted p-values, which were computed using the Statsmodels (v0.14.5) Stats library. Data visualization was done primarily in Python using the Matplotlib (v3.10.6) pyplot package. Violin plots were done using Seaborn (v0.13.2), and UpSet plots (13) were done in R (v4.5.1) using the UpSetR (v1.4.0) (14) package.

**SUPPLEMENTAL TEXT REFERENCES**

1. Howard-Varona C, Solonenko NE, Burris M, Urvoy M, Sanderson CM, Bolduc B, Sullivan MB. 2025. Infection and Genomic Properties of Single- and Double-Stranded DNA Cellulophaga Phages. Viruses 17.

2. Batth TS, Tollenaere MX, Ruther P, Gonzalez-Franquesa A, Prabhakar BS, Bekker-Jensen S, Deshmukh AS, Olsen JV. 2019. Protein Aggregation Capture on Microparticles Enables Multipurpose Proteomics Sample Preparation. Mol Cell Proteomics 18:1027–1035.

3. Sayers EW, Beck J, Bolton EE, Brister JR, Chan J, Connor R, Feldgarden M, Fine AM, Funk K, Hoffman J, Kannan S, Kelly C, Klimke W, Kim S, Lathrop S, Marchler-Bauer A, Murphy TD, O'Sullivan C, Schmieder E, Skripchenko Y, Stine A, Thibaud-Nissen F, Wang J, Ye J, Zellers E, Schneider VA, Pruitt KD. 2025. Database resources of the National Center for Biotechnology Information in 2025. Nucleic Acids Res 53:D20–D29.

4. Mirdita M, Schutze K, Moriwaki Y, Heo L, Ovchinnikov S, Steinegger M. 2022. ColabFold: making protein folding accessible to all. Nat Methods 19:679–682.

5. Jumper J, Evans R, Pritzel A, Green T, Figurnov M, Ronneberger O, Tunyasuvunakool K, Bates R, Zidek A, Potapenko A, Bridgland A, Meyer C, Kohl SAA, Ballard AJ, Cowie A, Romera-Paredes B, Nikolov S, Jain R, Adler J, Back T, Petersen S, Reiman D, Clancy E, Zielinski M, Steinegger M, Pacholska M, Berghammer T, Bodenstein S, Silver D, Vinyals O, Senior AW, Kavukcuoglu K, Kohli P, Hassabis D. 2021. Highly accurate protein structure prediction with AlphaFold. Nature 596:583–589.

6. Evans R, O’Neill M, Pritzel A, Antropova N, Senior A, Green T, Žídek A, Bates R, Blackwell S, Yim J, Ronneberger O, Bodenstein S, Zielinski M, Bridgland A, Potapenko A, Cowie A, Tunyasuvunakool K, Jain R, Clancy E, Kohli P, Jumper J, Hassabis D. 2022. Protein complex prediction with AlphaFold-Multimer. bioRxiv.

7. Zhang C, Shine M, Pyle AM, Zhang Y. 2022. US-align: universal structure alignments of proteins, nucleic acids, and macromolecular complexes. Nat Methods 19:1109–1115.

8. Pettersen EF, Goddard TD, Huang CC, Meng EC, Couch GS, Croll TI, Morris JH, Ferrin TE. 2021. UCSF ChimeraX: Structure visualization for researchers, educators, and developers. Protein Sci 30:70–82.

9. UniProt C. 2025. UniProt: the Universal Protein Knowledgebase in 2025. Nucleic Acids Res 53:D609–D617.

10. Song H, Parsons MR, Rowsell S, Leonard G, Phillips SE. 1999. Crystal structure of intact elongation factor EF-Tu from Escherichia coli in GDP conformation at 2.05 A resolution. J Mol Biol 285:1245–56.

11. Leone P, Roche J, Vincent MS, Tran QH, Desmyter A, Cascales E, Kellenberger C, Cambillau C, Roussel A. 2018. Type IX secretion system PorM and gliding machinery GldM form arches spanning the periplasmic space. Nat Commun 9:429.

12. Jones P, Binns D, Chang HY, Fraser M, Li W, McAnulla C, McWilliam H, Maslen J, Mitchell A, Nuka G, Pesseat S, Quinn AF, Sangrador-Vegas A, Scheremetjew M, Yong SY, Lopez R, Hunter S. 2014. InterProScan 5: genome-scale protein function classification. Bioinformatics 30:1236–40.

13. Lex A, Gehlenborg N, Strobelt H, Vuillemot R, Pfister H. 2014. UpSet: Visualization of Intersecting Sets. IEEE Trans Vis Comput Graph 20:1983–92.

14. Conway JR, Lex A, Gehlenborg N. 2017. UpSetR: an R package for the visualization of intersecting sets and their properties. Bioinformatics 33:2938–2940.
