# Supplementary material for: Detection and characterization of protein methylation in bacteriophages and their host, Cellulophaga baltica, during infection
Source: mSystems. 2026 May 18;11(6):e00012-26. doi: 10.1128/msystems.00012-26 (PMC13288928; doi:10.1128/msystems.00012-26)
Supplement: Reviewer comments [file reviewer-comments.pdf]

AMERICAN SOCIETY FOR MICROBIOLOGY | mSystems®

# Detection and Characterization of Protein Methylation in Bacteriophagesand Their Host, *Cellulophaga baltica,* During Infection

Andrew Stai, Cristina Howard-Varona3, Marion Urvoy, Marissa Gittrich, Matthew Sullivan, and Robert Hettich

*Corresponding Author(s): Robert Hettich, Oak Ridge National Laboratory*



## Review Timeline:

| | |
|---|---|
| Submission Date: | January 5, 2026 |
| Editorial Decision: | February 7, 2026 |
| Revision Received: | April 6, 2026 |
| Accepted: | April 17, 2026 |

*Editor: Jyot Antani*

*Reviewer(s): Disclosure of reviewer identity is with reference to reviewer comments included in decision letter(s). The following individuals involved in review of your submission have agreed to reveal their identity: Rui Zhang (Reviewer #1); Wei-Hua Chen (Reviewer #3); Marco Rinaldo Oggioni (Reviewer #4)*

## Transaction Report:

(Note: With the exception of the correction of typographical or spelling errors that could be a source of ambiguity, letters and reports are not edited. The original formatting of letters and referee reports may not be reflected in this compilation.)

**DOI: https://doi.org/10.1128/msystems.00012-26**

Re: mSystems00012-26 (**Detection and Characterization of Protein Methylation in Bacteriophages and Their Host, *Cellulophaga baltica,* During Infection**)

Dear Dr. Robert L Hettich,

Thank you for the privilege of reviewing your work. Below you will find my comments, instructions from the mSystems editorial office, and the reviewer comments.

Congratulations on submitting a well-written manuscript with carefully designed experiments and clear results. Please address the reviewers' comments to lightly edit and thus further strengthen the already strong manuscript. Also, please ensure that the dataset is publicly available on both the MassIVE repository (it is currently password-protected for reviewers) and ProteomeXchange (where it is not yet available).

Please return the manuscript within 60 days; if you cannot complete the modification within this time period, please contact me. If you do not wish to modify the manuscript and prefer to submit it to another journal, notify me immediately so that the manuscript may be formally withdrawn from consideration by mSystems.

**Revision Guidelines**
To submit your modified manuscript, log into the submission site at https://msystems.msubmit.net/cgi-bin/main.plex. Go to Author Tasks and click the appropriate manuscript title to begin. The information you entered when you first submitted the paper will be displayed; update this as necessary. Note the following requirements:

• Upload point-by-point responses to the issues raised by the reviewers in a file named "Response to Reviewers," NOT in your cover letter.
• Upload a compare copy of the manuscript (without figures) as a "Marked-Up Manuscript" file.
• Upload a clean .DOC/.DOCX version of the revised manuscript and remove the previous version.
• Each figure must be uploaded as a separate, editable, high-resolution file (TIFF or EPS preferred), and any multipanel figures must be assembled into one file.
• Any supplemental material intended for posting by ASM should be uploaded with their legends separate from the main manuscript. You can combine all supplemental material into one file (preferred) or split it into a maximum of 10 files with all associated legends included.

For complete guidelines on revision requirements, see our Submission and Review Process webpage. Submission of a paper that does not conform to guidelines may delay acceptance of your manuscript.

**Data availability:** ASM policy requires that data be available to the public upon online posting of the article, so please verify all links to sequence records, if present, and make sure that each number retrieves the full record of the data. If a new accession number is not linked or a link is broken, provide mSystems production staff with the correct URL for the record. If the accession numbers for new data are not publicly accessible before the expected online posting of the article, publication may be delayed; please contact production staff (mSystems@asmusa.org) immediately with the expected release date.

**Publication Fees:** For information on publication fees and which article types are subject to charges, visit our website. If your manuscript is accepted for publication and any fees apply, you will be contacted separately about payment during the production process; please follow the instructions in that e-mail. Arrangements for payment must be made before your article is published.

**ASM Membership:** Corresponding authors may join or renew ASM membership to obtain discounts on publication fees. Need to upgrade your membership level? Please contact Customer Service at Service@asmusa.org.

The ASM Journals program strives for constant improvement in our submission and publication process. Please tell us how we can improve your experience by taking this quick Author Survey.

Thank you for submitting your paper to mSystems.

Sincerely,
Jyot Antani, PhD

Reviewer #1 (Comments for the Author):

This study provides a comprehensive study of the roles of protein methylation in both bacteriophage and host cell proteins during phage-host interactions. I have some comments (listed below) that may help the authors further improve the quality of the manuscript.

Lines 192-200: The explanation for the pattern of phi18:1 MCP K44 dimethylation should be discussed in conjunction with the one-step growth curve of phi18:1. It might be helpful to include this curve in Figure 2A for comparison.

Lines 200-204: This section does not clearly illustrate the role of phage protein methylation in the phage-host interaction. Is there no connection between methylation on phage proteins and the host's methylation response to phage infection?

Lines 210-214: Regarding the statement "some methylation events were only found early in infection and were not detected in the late stages of infection": Why are these methylation events not maintained on the phage proteins? Could the host encode and express proteins with demethylase activity?

Lines 266-270: Why does the same bacterial strain employ different methylation response strategies against different phages?

Lines 314-316: How does EF-Tu methylation lead to attenuated GTP hydrolysis? In E. coli or other organisms, are the methylation sites on EF-Tu also spread spatially throughout the protein?

Lines 333-336: What evidence suggests that phi18:1 infection inhibits the stress response of the host cells? Burst size may not be direct evidence for this claim.

Lines 391-393: Do the methylation patterns of DnaK and the OMP in phi18:1 virocells show changes similar to those described for the phi18:1 infection cycle in lines 192-200?

Reviewer #3 (Comments for the Author):

This manuscript reports a time-resolved, MS-based proteomic analysis of protein methylation during infection of Cellulophaga baltica by three distinct bacteriophages.

The authors present evidence that protein methylation is globally elevated in virocells relative to uninfected controls and, notably, that phage-encoded proteins themselves can be methylated, with some modifications persisting into free virions. The dataset is extensive, the experimental system is well chosen, and the work addresses an underexplored post-translational modification in phage-host interactions. Overall, the study is potentially impactful and well aligned with the scope of mSystems, but several technical and interpretative limitations temper the strength of the conclusions.

Major concerns:

1. The results are mostly descriptive, with providing solid evidence to support the regulatory roles of the methylated proteins by perturbation experiments.

2. The confidence in methylation site assignment might be low. Particularly, while high-resolution LC-MS/MS is a well-established approach for detecting protein methylation, methylation remains a chemically subtle modification (+14 Da per methyl group) that can be confounded with amino acid substitutions, missed cleavages, or other small mass shifts. The study relies on untargeted DDA data without orthogonal validation, limits confidence in site-level assignments, particularly for low-abundance peptides and multiply methylated residues.

3. Lack of independent validation of key methylation events. The authors highlighted examples of protein methylation (e.g., EF-Tu, DnaK, major capsid proteins) and putative regulatory roles. However, although these claims are biologically plausible and intriguing, no independent biochemical or genetic validation is provided.

4. The results might be biased by protein abundance changes. Particularly, the authors acknowledge that methylpeptide detection is strongly correlated with protein abundance. This raises the possibility that the observed increase in methylation during infection may partly reflect infection-driven changes in protein abundance or detectability rather than true increases in modification stoichiometry. Without normalization to site occupancy or targeted quantification, conclusions about "increased methylation" should be interpreted cautiously.

Overall, the study provides the first systematic view of protein methylation dynamics during phage infection and opens an interesting new line of inquiry in virocell biology. However, the conclusions-especially regarding phage protein methylation and functional relevance-would be strengthened by additional validation, clearer acknowledgment of MS-specific limitations, and a more restrained interpretation of causality. With these caveats addressed, the work would represent a solid and novel contribution to the field.

Reviewer #4 (Comments for the Author):

A very nice paper describing an underinvestigated aspect of microbial life. The data are well reported, even if I am unable to judge the methodological accuracy of protein methylation reported.

One of the main questions I would have to the paper is if the methylation is phage or host driven. In order to understand if the protein methylation is host or phage driven, it would be interesting to test if a classical stress also linked to induction of phage (i.e. UV) could lead to the same increase in protein methylation. Also some comments on the presence of potential methyltransferases in the phage genomes would be helpful.

The manuscript by Stai and co-workers examines protein methylation of the marine bacterium *Cellulophaga baltica* from control and phage-infected conditions with bacteriophage that differ in their ability to efficiently infect *C. baltica.* Ultimately, the data indicate methylation is occurring during bacteriophage infection and that both the host and phage proteins are subject to methylation. The text reads more speculative when discussing the function of these methylation events. Without more mechanistic experiments, several of the conclusions feel uncomfortable, even framed as speculation with comparative analyses. Please refer below to suggestions regarding the manuscript.

**Major Comments**
The methods used to produce the date in Table 1 needs to be re-addressed. The authors measured methylated peptides in four sets of cultures (mock infection and three phage infections) over time in four biological replicates. First, there appears to be significant variation in the methylation profile within the untreated cultures over time in the experiment. This may be noise, but it should be addressed as some of the infected cultures also have similar methylation patterns over time. Second, the method used to analyze the difference in the average methylated peptides per condition should be reconsidered. Pooling all samples for uninfected (n = 54) and infected (n = 24) leads to pseudoreplication, as each individual sample is not independent. Instead, each replicate should be considered over time (i.e., n = 4) for each phage infection and analyzed with a time-weighted mean.

Signal intensity should be clearly defined within the text given the amount it is used. Additionally, the definition is vague and lacks description of how it is calculated. When possible, the fraction of methylated peptides per total peptides should be included. Without this, it is very difficult to get a sense of the quantitative nature of the methylation events.

Line 238 “targeted for methylation” is too strong of wording given the data. Targeted implies they are “chosen” because of their function when a more parsimonious explanation is that they are methylated because of their abundance.

Line 264 – Is it possible that the abundance of Ef-Tu in the other infections masks the detection of less abundant methylated peptides. Thus, these methylated events in phi18:1 may not be unique to phi18:1.

Line 271 – Header needs to be changed to reflect the comparative and speculative nature of the analysis. It is best to stick with what the analysis identified “Elongation factor-Tu Protein is Prevalently and Variably Methylated after Phage Infection”.

Line 371 – It would be helpful if Figure 5 was more quantitative. The heatmap displays presence/absence, but of the “present” population, what fraction of DnaK peptides measured were methylated?

Line 410 – If there were no phage dependent methylations in the gliding proteins, how can one speculate on potential functions during phage infection? This contradiction

should be clarified. Additionally, the use of “targeted” (Line 426) over-states the data presented in the paragraph, particularly the lack of a trend with the conditions test.

References 62 and 66 are unavailable as pre-prints and are under-review. Reference 66 contains the methodology used to culture and sample the virocell and uninfected control samples. This information should be explicitly included in the main text or the supplemental text if space is an issue.

**Response to Reviewers**

Reviewer #1 (Comments for the Author):

This study provides a comprehensive study of the roles of protein methylation in both bacteriophage and host cell proteins during phage-host interactions. I have some comments (listed below) that may help the authors further improve the quality of the manuscript.

Lines 192-200: The explanation for the pattern of phi18:1 MCP K44 dimethylation should be discussed in conjunction with the one-step growth curve of phi18:1. It might be helpful to include this curve in Figure 2A for comparison.

Response #1: As all of the sampling was done in the latent period of phage infection, very little increase in PFUs was detected within the 0-45 minutes post-dilution time frame shown in Figure 2A. The relatively small change in PFUs is illustrated in Figure 4 of this paper (https://www.mdpi.com/1999-4915/17/3/365) (1), where there is no large increase in PFUs until ~65 minutes, which is after our last sampled time point for phi18:1. Therefore, the one-step growth curve line would have been primarily flat for the entirety of Figure 2A and, after some discussion amongst the authors, we felt that including it would not have enhanced the figure or the discussion in that section. However, we agree with the Reviewer that the one-step growth curves and information on latent periods are valuable information to include, and so this information and their references have been added to the very beginning of the Results and Discussion section on lines 134-137.

Lines 200-204: This section does not clearly illustrate the role of phage protein methylation in the phage-host interaction. Is there no connection between methylation on phage proteins and the host's methylation response to phage infection?

Response #2: To address the reviewer concern here, we have expanded upon that discussion of the possible role the K44 dimethylation on phi18:1's MCP may play in the interaction between the phage and host in lines 205-208.

As for the connection between methylation on phage proteins and the host's methylation response, we concur that this is an important topic but one for which there is relatively little that is understood. Our primary focus here was an elucidation of the phage-specific and temporal dynamics of protein methylation, both in phage and host. This now provides a broad foundation of information about modified proteins as a function of infection conditions. While we agree that an investigation of the connection of phage protein methylation and host methylation response is interesting, this was unable to be executed in our current experimental design and thus felt it

would be managed better in a focused, subsequent study. We have edited the end of the Conclusions section in lines 457-461 to highlight the importance of protein-specific mechanistic study in the next steps of this line of inquiry.

Lines 210-214: Regarding the statement "some methylation events were only found early in infection and were not detected in the late stages of infection": Why are these methylation events not maintained on the phage proteins? Could the host encode and express proteins with demethylase activity?

Response #3: We agree that the host encoding a demethylase is likely, and this may be the reason these methylation events are not maintained on the phage proteins. None of the current gene annotations indicate the presence of a demethylase in the Cba18 genome. However, a large portion of the host proteins detected in any sample (653/3,334) had no annotation, so we cannot confidently say this organism is not synthesizing demethylases. To investigate this further and address the reviewer concern, we used the NCBI BLAST feature to search for a protein in the Cba18 genome displaying sequence similarity to a predicted lysine demethylase from another bacterium in the same family as Cba18 (UniProt ID A0ABM9NWM9_9FLAO https://www.uniprot.org/uniprotkb/A0ABM9NWM9/entry). This yielded a 77% amino acid identity match with the expressed Ga0325142_111104, which is annotated as being a cupin-like protein. There is a family of histone demethylases that have been found to belong to the cupin superfamily (https://pmc.ncbi.nlm.nih.gov/articles/PMC2579438/) (2), so this protein may very well represent a functional demethylase in the Cba18 genome.

Another alternative explanation is that at least some of these disappearing methylation events may be due to protein degradation/turnover, where methylated proteins are being degraded and, if the protein is still needed by the phage for infection, being synthesized anew without any methylation. Our experimental approach was not designed to address this latter explanation.

Both of these explanations have been added to the manuscript in lines 218-230 to enhance the discussion.

Lines 266-270: Why does the same bacterial strain employ different methylation response strategies against different phages?

Response #4: Two likely explanations here are that the different phenotypes of the phages (e.g., ssDNA vs. dsDNA, tailed vs. non-tailed, etc.) activate different responses of the bacterium or that these differing methylation responses are phage-driven and not host-driven.

Lines 314-316: How does EF-Tu methylation lead to attenuated GTP hydrolysis? In E. coli or other organisms, are the methylation sites on EF-Tu also spread spatially throughout the protein?

Response #5: The assertion that EF-Tu methylation may lead to an attenuated GTP hydrolysis comes from this publication (https://febs.onlinelibrary.wiley.com/doi/10.1111/j.1432-1033.1986.tb10074.x) (3). This paper showed that when K56 is methylated, EF-Tu's GTPase

activity is inhibited compared to the non-K56 methylated version of EF-Tu. The authors hypothesized that the GTP hydrolysis step is inhibited because they saw no effect of methylation on the dissociation of GDP from EF-Tu or on the ability of EF-Tu to bind to its tRNA inducer; however, they did not have a tested explanation for why the inhibition occurs. Their hypothesis was that the tRNA-induced "conformational effects on EF-Tu are affected by the methylation of the protein".

As for the spatial distribution of EF-Tu methylation sites in other organisms, much of the work looking at EF-Tu methylation is old, when determining the location of methylation events with the currently available techniques was more difficult and labor-intensive. Van Noort et al. only found EF-Tu to be methylated at a single residue (K56) in *E. coli*. Several other groups that have found EF-Tu methylation did not examine the location of this/these methylation events.

We acknowledge the need for an improved explanation of EF-Tu methylation leading to attenuated GTPase activity and so have inserted a sentence in lines 304-305 explaining this hypothesis as well as edited the surrounding sentences to improve clarity.

Lines 333-336: What evidence suggests that phi18:1 infection inhibits the stress response of the host cells? Burst size may not be direct evidence for this claim.

Response #6: As the Reviewer points out, the claim that phi18:1 may inhibit the stress response of Cba18 cells comes from the reduction of EF-Tu K57 methylation in phi18:1 virocells being observed concomitantly with an increase in burst size, with this explanation given in lines 348-352. EF-Tu methylation has known ties to the stress response of *E. coli*, as it has been previously seen during the nutrient deprivation stress response (https://journals.asm.org/doi/10.1128/jb.173.10.3096-3100.1991) (4). Therefore, as EF-Tu K57 methylation is reduced at T0 in phi18:1 virocells, we propose that this may be part of an effort by phi18:1 to prevent a stress-induced reduction in protein synthesis, enabling it to replicate unimpeded.

We have modified the manuscript at lines 332-333 and 356-357 to make this explanation clearer.

Lines 391-393: Do the methylation patterns of DnaK and the OMP in phi18:1 virocells show changes similar to those described for the phi18:1 infection cycle in lines 192-200?

Response #7: Yes, both methylation events on DnaK and the OMP disappear at T30 like the MCP. This aligns with the wider decrease in identified methylpeptides at T30 in phi18:1 virocells that is shown in Table 1, where this time point had the fewest methylpeptides identified of any time point in any of the three infections. While the cause of this large reduction in methylpeptide identifications is unknown, we believe that this is a valuable observation and have added this to the manuscript on lines 390-392.

Reviewer #3 (Comments for the Author):

This manuscript reports a time-resolved, MS-based proteomic analysis of protein methylation during infection of Cellulophaga baltica by three distinct bacteriophages.

The authors present evidence that protein methylation is globally elevated in virocells relative to uninfected controls and, notably, that phage-encoded proteins themselves can be methylated, with some modifications persisting into free virions. The dataset is extensive, the experimental system is well chosen, and the work addresses an underexplored post-translational modification in phage-host interactions. Overall, the study is potentially impactful and well aligned with the scope of mSystems, but several technical and interpretative limitations temper the strength of the conclusions.

Thank you for these positive, supportive comments; we will address each of the concerns specifically below.

Major concerns:

1. The results are mostly descriptive, with providing solid evidence to support the regulatory roles of the methylated proteins by perturbation experiments.

Response #8: While we acknowledge the reviewer's comment here, we would emphasize that our results lay important groundwork about the nature of bacterial-phage methylation connections.  Our objective here was to systematically examine the range and dynamics of both phage and host protein methylation during phage infection, the former of which to our knowledge has never been described before. Herein we report phage protein methylation, discuss possible structural or regulatory roles, and identify carry-over into the virion. Host methylation has never been compared across infections with multiple phages differing in efficiency and genomic properties. As such, this work provides the first global view of methylation dynamics across both host and phage proteins during infection, a previously unexplored regulatory layer in virocells. This allowed us to clearly identify candidate proteins and pathways who represent promising targets for future mechanistic investigation.

While functionally exploring mechanisms is an important next step, we feel that it is beyond the scope of this manuscript. We have now better clarified this point in the introduction and conclusion of the manuscript and emphasize that the results presented here establish a foundational framework and resource that enables future experimental interrogation of methylation-mediated regulation during phage infection (lines 113 and 457-461).

2. The confidence in methylation site assignment might be low. Particularly, while high-resolution LC-MS/MS is a well-established approach for detecting protein methylation, methylation remains a chemically subtle modification (+14 Da per methyl group) that can be confounded with amino acid substitutions, missed cleavages, or other small mass shifts. The study relies on untargeted DDA data without orthogonal validation, limits confidence in site-level assignments, particularly for low-abundance peptides and multiply methylated residues.

Response #9: We agree with the Reviewer that correct localization of methylation on peptides carries extra difficulty but would contend that, with careful analysis, much of this can be addressed. For example, we believe that this challenge was somewhat alleviated by our automatic searches for methylation being restricted to arginines and lysines. As we used trypsin for proteolytic cleavage, which primarily cuts on the C-terminal end of lysines and arginines, the vast majority of our peptides had their methylation predicted to be located on the arginine or lysine on the C-terminal end of the peptide. Given the prevalence of *y*-type ions in fragmentation spectra of peptides, this allowed us to nearly always have a $y_1$ or $y_2$ ion peak with the expected mass shift corresponding to the number of methyl groups added to the peptide, providing evidence for localization of the methylation event down to, at the very least, the last two residues on the C-terminal end. As this is a legitimate concern in mass spectrometry-based PTM analysis, we felt that this additional information would prove useful to the reader and so have added a paragraph beginning with "Although mass spectrometry…" in the "Peptide identification and data analysis" section of the Supplemental Text following where we provide an in-depth discussion of our methylation detection and manual validation.

Additionally, to address specifically the concern of amino acid substitutions mimicking a methylation event, we found that the majority of methylpeptides identified (1,122 out of 1,487 total) had an unmodified version of the peptide also identified. The existence of an unmodified peptide of the same sequence as a proposed methylpeptide makes the likelihood of an amino acid substitution being responsible for the identification less likely, as it would necessitate only a portion of the population containing the amino acid substitution, but also that portion being of a sizeable enough amount to allow for identification of the variant peptide.

Furthermore, we believe our identifications are further strengthened by our manual inspection of the fragmentation spectra of all methylation events specifically named in this work (i.e., any time a methylation event was referred to by its residue and protein). The criteria used for manually validating a methylation event are provided in the Supplemental Text under the "Peptide identification and data analysis" subsection, but we have copied them below for ease of review:

> "All methylation events specifically named in this work were manually validated by inspection of MS2 fragmentation spectra in Proteome Discoverer, resulting in visual inspection of ~190 methylpeptides' MS2 spectra in total. Spectra complexity, retention time shift, peak intensities, and proportion of peptide sequence covered by *b* and *y* ions all were used in manual validation. Additionally, if unmodified versions of the peptide

were detected, the relative intensity of the peaks of the suspected methylated peptide were compared to the relative intensity of the peaks of the unmethylated peptide of the same charge state. Methylpeptides were considered true positives if: (i) they fell within a range of retention time shifts expected of the addition of one or more methyl groups, (ii) the majority of the peptide's residues had representative gaps between peaks corresponding to their mass, (iii) the spectra was either primarily composed of peaks that matched the peptide sequence or had explainable non-matching peaks, (iv) the relative intensity of the peaks in the methylpeptide matched the relative intensity of the peaks in the unmethylated peptide of the same charge state, if available."

3. Lack of independent validation of key methylation events. The authors highlighted examples of protein methylation (e.g., EF-Tu, DnaK, major capsid proteins) and putative regulatory roles. However, although these claims are biologically plausible and intriguing, no independent biochemical or genetic validation is provided.

Response #10: As stated above in Response #8, we feel that the strength of this study is the broad but detailed elucidation of both phage and host protein methylation in a dynamic context. As this has never been characterized previously, we felt that it was important to evaluate the range, dynamics, and identities of proteome methylation here at an untargeted global proteome scale. In order to demonstrate some specific biological connections, we focused on several key, "suspected" phage-related host proteins. We concur that further examination of biological or genetic validation would be informative but this was beyond the scope of this report.

4. The results might be biased by protein abundance changes. Particularly, the authors acknowledge that methylpeptide detection is strongly correlated with protein abundance. This raises the possibility that the observed increase in methylation during infection may partly reflect infection-driven changes in protein abundance or detectability rather than true increases in modification stoichiometry. Without normalization to site occupancy or targeted quantification, conclusions about "increased methylation" should be interpreted cautiously.

Response #11: We appreciate the Reviewer's concern, as we shared this concern ourselves when analyzing and interpreting the data. We believe there are several lines of evidence that support the increase in methylation not being driven by an increase in protein abundance or detectability. First, in regards to the increased number of methylpeptides identified in phage infection as shown in Table 1, in Supplemental Text under the "Protein extraction and liquid chromatography…" subsection, we outline our approach for normalizing the amount of protein loaded in each sample by measuring peptide concentration at the end of extraction, then using those values to target a loading amount of 3 µg of peptides:

"Peptides were then filtered using a 10 kDa MWCO filter plate (AcroPrep Advance, Omega 10 K MWCO) and quantified again using a NanoDrop OneC. For each sample, 3 µg of peptides were loaded onto a liquid chromatography tandem mass spectrometry

(LC-MS/MS) setup using a Vanquish ultra-HPLC system coupled with a QExactive-Plus mass spectrometer (Thermo Fisher Scientific) (Figure 1C)."

Infection-driven changes in overall protein abundance should mostly have been negated by this step. Secondly, Supplementary Table 1 demonstrates that we identified more proteins in the uninfected cells than in the phage-infected cells. If the increase in methylpeptide detection in virocells was a result of increased protein detectability as a whole, then we would have expected there to be more proteins detected in the three virocell conditions and not in the uninfected cells. Finally, in regards to the increased host methylation levels at T0 shown in Figure 3B, these levels are normalized by total protein abundance, as the values are the percent of the total signal intensity from all measured proteins attributed to the methylpeptides. If increased protein abundance as a whole was driving this observed increase in host methylation levels, the percent of signal intensity coming from methylpeptides would not be expected to change as much. What Figure 3B demonstrates is that the portion of the total protein signal that is coming from methylpeptides is significantly increased at T0 for the phi38:1 and phi18:4 virocells. We have clarified how the values were calculated in Figure 3B in the figure caption.

Overall, the study provides the first systematic view of protein methylation dynamics during phage infection and opens an interesting new line of inquiry in virocell biology. However, the conclusions-especially regarding phage protein methylation and functional relevance-would be strengthened by additional validation, clearer acknowledgment of MS-specific limitations, and a more restrained interpretation of causality. With these caveats addressed, the work would represent a solid and novel contribution to the field.

We thank the Reviewer for their comments and concerns and hope that we have adequately addressed them and clarified our intention with this work, both in these responses and in our edits to the manuscript.

Reviewer #4 (Comments for the Author):

A very nice paper describing an underinvestigated aspect of microbial life. The data are well reported, even if I am unable to judge the methodological accuracy of protein methylation reported.

One of the main questions I would have to the paper is if the methylation is phage or host driven. In order to understand if the protein methylation is host or phage driven, it would be interesting to test if a classical stress also linked to induction of phage (i.e. UV) could lead to the same increase in protein methylation. Also some comments on the presence of potential methyltransferases in the phage genomes would be helpful.

We agree with the Reviewer that the question of methylation being host or phage driven is of great interest and appreciate the suggestion of a potential experiment to help decipher this. After discussing the benefits of carrying out this experiment among the authors, we believe that this experiment of applying a classical stress would ultimately not resolve the issue of determining which entity is responsible for driving the methylation. As bacteria's responses to stress can be quite stressor-specific (e.g., different stress responses to DNA damage vs. nutrient deprivation), the absence of a methylation event in a classical stress experiment would not confirm that the methylation event had been phage-driven in the infection, as that may have been a response of the bacterium specific to that phage. Additionally, it is worth pointing out that Cba18 is not predicted to have any prophages, and so this classical stress would not be inducing any phages and therefore would be causing a different kind of stress than that caused by phage infection.

However, we did attempt to address this question of phage or host driven methylation with the given data by investigating methylation events that only appeared in phage-infected virocells but were missing in uninfected cells (discussed in "Several Methylation Events on Proteins…" subsection in Results). While these shared methylation events may be phage-driven and a common technique utilized by the phages to take over the host, we believe that is it much more likely that are host-driven responses to the different phages. We note that the original wording on line 375 suggested that these methylation events were part of a general host response and have edited this sentence as well as expanded the concluding sentence of the section on lines 410-411 to clarify that these may be host or phage driven methylation events.

As for the presence of methyltransferases in the phage genome, there are no predicted methyltransferases in the phage genome. The submitted manuscript did have a sentence on line 211 detailing this, but the wording was slightly ambiguous and so it has been edited to clarify this point.

## References


1. Howard-Varona C, Solonenko NE, Burris M, Urvoy M, Sanderson CM, Bolduc B, et al. Infection and Genomic Properties of Single- and Double-Stranded DNA Cellulophaga Phages. Viruses. 2025;17(3).
2. Zhou X, Ma H. Evolutionary history of histone demethylase families: distinct evolutionary patterns suggest functional divergence. BMC Evol Biol. 2008;8:294.
3. Van Noort JM, Kraal B, Sinjorgo KM, Persoon NL, Johanns ES, Bosch L. Methylation in vivo of elongation factor EF-Tu at lysine-56 decreases the rate of tRNA-dependent GTP hydrolysis. Eur J Biochem. 1986;160(3):557–61.
4. Young CC, Bernlohr RW. Elongation factor Tu is methylated in response to nutrient deprivation in Escherichia coli. J Bacteriol. 1991;173(10):3096–100.

Re: mSystems00012-26R1 (**Detection and Characterization of Protein Methylation in Bacteriophages and Their Host, *Cellulophaga baltica,* During Infection**)

Dear Dr. Robert L Hettich:

Congratulations!

Your manuscript has been accepted, and I am forwarding it to the ASM production staff for publication. Your paper will first be checked to make sure all elements meet the technical requirements. ASM staff will contact you if anything needs to be revised before copyediting and production can begin. Otherwise, you will be notified when your proofs are ready to be viewed.

**Data Availability:** ASM policy requires that data be available to the public upon online posting of the article, so please verify all links to sequence records, if present, and make sure that each number retrieves the full record of the data. If a new accession number is not linked or a link is broken, provide production staff with the correct URL for the record. If the accession numbers for new data are not publicly accessible before the expected online posting of the article, publication may be delayed; please contact ASM production staff immediately with the expected release date.

**Publication Fees:** For information on publication fees and which article types have charges, please visit our website. We have partnered with Copyright Clearance Center (CCC) to collect author charges. If fees apply to your paper, you will receive a message from no-reply@copyright.com with further instructions. For questions related to paying charges through RightsLink, please contact CCC at ASM_Support@copyright.com or toll free at +1-877-622-5543. CCC makes every attempt to respond to all emails within 24 hours.

**ASM Membership:** Corresponding authors may join or renew ASM membership to obtain discounts on publication fees. Need to upgrade your membership level? Please contact Customer Service at Service@asmusa.org.

**Embargo Policy:** A press release may be issued as soon as the manuscript is posted on the mSystems Latest Articles webpage. The corresponding author will receive an email with the subject line "ASM Journals Author Services Notification" when the article is available online.

**Cover Image Submissions:** If you would like to submit a potential Cover Image, please email a file and a short legend to msystems@asmusa.org. Please note that we can only consider images that (i) the authors created or own and (ii) have not been previously published. By submitting, you agree that the image can be used under the same terms as the published article. Image File requirements: TIF/EPS, 7.5 inches wide by 8.25 inches tall (at least 2,250 pixels wide by 2,475 pixels tall), minimum 300 dpi resolution (600 dpi preferred), RGB, and no figure elements, e.g., arrows or panel labels. The legend should be a short description of the image, 1-2 sentences recommended. Please download and use this interactive template in Adobe to ensure that your proposed cover image meets our size requirements (https://journals.asm.org/pb-assets/pdf-text-excel-files/ASM-Interactive-Sizing-Cover-Template-1715689791.pdf).

Thank you for submitting your paper to mSystems.

Sincerely,
Jyot Antani, PhD

Reviewer #1 (Comments for the Author):

The authors have carefully addressed my previous comments and have made appropriate revisions to the manuscript. The additional clarifications and expanded discussions have improved the overall quality and clarity of the work. I have no further major concerns, and I believe the manuscript is now suitable for publication.

Reviewer #3 (Comments for the Author):

all my previous concerns have been adequately addressed.
